# A Systematic Review and Meta‐Analysis of Randomized Controlled Trials of Behavioral Interventions for the Management of Overweight and Obesity in Children That Are Delivered or Referred to by Health Providers in Primary Care

**DOI:** 10.1111/obr.70119

**Published:** 2026-03-10

**Authors:** Henrietta E. Graham, Claire D. Madigan, Kajal Gokal, Jessica F. Large, James Sanders, Chris J. McLeod, Natalie Pearson, Amanda J. Daley

**Affiliations:** ^1^ Centre for Lifestyle Medicine and Behaviour (CLiMB), The School of Sport, Exercise and Health Sciences Loughborough University Loughborough UK

**Keywords:** behavioral weight management, children, obesity

## Abstract

**Introduction:**

Reducing childhood overweight and obesity prevalence is a global public health priority. This systematic review and meta‐analysis evaluated the effectiveness of behavioral weight management interventions delivered or referred to by health care providers in primary care settings.

**Methods:**

Randomized controlled trials (RCTs) of behavioral interventions published up to 05/01/2026, involving participants < 18 years with overweight or obesity, were identified through Cochrane, MEDLINE, PubMed, and PsychINFO. Two reviewers independently screened studies, extracted data, and assessed risk of bias. Meta‐analyses calculated pooled mean differences in zBMI and BMI using random effects models. The primary outcome was zBMI change at 12 months; secondary outcomes included zBMI changes at program end, last follow‐up, and BMI at 12 months.

**Results:**

Fifty‐nine RCTs (*n* = 10,454) were included; 23 trials (*n* = 3241) contributed to the primary outcome. At 12 months, the pooled mean difference in zBMI was −0.08 (95% CI −0.13 to −0.03, *p* < 0.01), favoring intervention groups. At program end (*n* = 30), the mean difference was −0.15 (95% CI −0.22 to −0.08), and at last follow‐up (*n* = 37), −0.08 (95% CI −0.15 to −0.02). BMI at 12 months showed a mean difference of −0.37 (95% CI −0.72 to −0.01). Interventions referred to community settings achieved greater zBMI reductions (−0.14 [95% CI −0.2 to −0.08]) than those delivered within primary care (0.04 [95% CI −0.10 to 0.18]).

**Conclusions:**

Behavioral weight management interventions for children delivered or referred to by health care professionals in primary care led to modest reductions in zBMI. Referrals to community‐based interventions (e.g., HENRY) may yield greater improvements.

## 1 Introduction

Approximately 37 million children under 5 years of age and 390 million children aged 5–19 years are living with overweight or obesity worldwide [1]. The prevalence of overweight and obesity in children has risen, from about 8% in 1990 to 20% in 2022 [1]. Obesity is a multifactorial disease attributed to energy imbalance, obesogenic environments, cultural and psycho‐social factors, and genetics [2]. Evidence suggests that prevalence is strongly correlated with socio‐economic status, with children living in the most deprived communities being more than twice as likely to be living with obesity than children in the least deprived areas [3]. Excess weight in childhood is a strong predictor for obesity in adulthood with an increased risk of developing chronic diseases such as type 2 diabetes, cardiovascular disease and poorer oral health [1, 4]. School performance and quality of life can also be negatively impacted, with experience of weight stigma commonly reported in children [5]. Given the adverse outcomes associated with obesity, health agencies and governments across the globe are directing considerable effort towards finding effective treatments to reduce obesity rates in children.

Children in high‐income countries typically attend routine appointments with their general practitioner/family doctor on several occasions throughout their childhood. Health providers working in primary care have a unique opportunity to intervene at several stages across the life course of children. A systematic review of RCTs published in 2016 (*n* = 10 trials) found that, compared to usual care, brief weight management interventions resulted in a small reduction in children's zBMI (−0.04, 95% CI −0.08 to −0.01) [6]. A review of RCTs by O'Conner et al. [7] found that weight management interventions (including prevention of obesity) in children that were conducted in, or participants were recruited from, healthcare settings resulted in a mean difference in BMI (kg/m^2^) of −0.7 [95% CI, −1.0 to −0.3] between 6 and 12 months after the start of the intervention. They also found that including physical activity sessions and more contact hours was associated with greater BMI change between 6 and 12 months after the start of the intervention, although these two factors could be interrelated. However, the review by O'Conner included studies that also focused on weight gain prevention, and trials with usual care above two annual contacts were excluded. This approach to usual care could be problematic because “usual care” in some countries might involve more than two contacts. Adopting this criterion may have inflated the effect size reported by the review. Additionally, the O'Conner review included trials with data between 6 and 12 months in the primary outcome analysis. When providing evidence of the effectiveness of behavioral weight management interventions, it is important to distinguish between shorter‐term (~6 months) and longer‐term follow‐up (~12 months) of outcomes. Combining the analysis of these time points may produce more favorable shorter‐term effects than would be the case if interventions were routinely implemented within healthcare over a longer time frame.

Building on and extending the previous systematic review by O'Conner and colleagues, the primary aim of this systematic review was to examine the effectiveness of behavioral weight management interventions for children (aged 0–18 years) that could be delivered or referred to by health professionals in primary care, at 12‐month follow‐up. Through predefined sub‐group analyses, a further aim was to investigate the contexts in which interventions may be most effective to optimize later implementation. Recognizing that children from lower socio‐economic backgrounds are more likely to experience obesity, and with a commitment to create a more inclusive society, the association of equality indicators (e.g., ethnicity and household income) on outcomes were examined to address the question of equity of interventions [8]. This review will also provide evidence to guide global policy decisions on implementing behavioral interventions in primary care to address rising childhood obesity rates.

## 2 Materials and Methods

The systematic review and meta‐analysis of randomized controlled trials (RCTs) was conducted according to PRISMA guidelines and was prospectively registered on PROSPERO (CRD42023437891).

### 2.1 Eligibility Criteria

RCTs with participants aged < 18 years that evaluated behavioral weight management interventions that could either be delivered or referred to by health care providers in primary care settings were eligible (i.e. available in the community). A primary care setting was broadly defined as the first point of contact with the healthcare system, providing accessible, continued, comprehensive, and coordinated care which focuses on the long‐term health of a person [9]. Table 1 details the full inclusion and exclusion criteria of eligible trials. Trials were included if they collected zBMI or BMI data.

**TABLE 1:** Inclusion and exclusion criteria.

	Inclusion	Exclusion
**Study aim**	Weight loss	Primary prevention of overweight or obesity Treatment of cardiovascular disease Treatment of cancer Weight loss maintenance
**Condition definition**	Overweight or obesity as defined by the study authors	
**Population**	Young people aged < 18 years who are candidates for weight loss interventions and selected based on living with overweight and obesity (as defined by the study authors)	Trials limited to: Populations not selected based on a weight‐related measure (i.e., BMI, waist circumference, weight) Children with secondary causes of obesity, such as steroid use, Prader Willi syndrome Children with a known chronic disease not generalizable to the primary care population (e.g., eating disorder, cancer, chronic kidney disease, severe mental illness, cognitive impairment) Adults
**Setting**	Intervention must have been conducted in primary care, feasible to be conducted in primary care, or comparable to programs widely available for referral from primary care. Primary care is defined as the first point of contact, based in the community, can offer ongoing and comprehensive healthcare	Settings not feasible for implementation in primary care or health care systems to which primary care providers could not refer, such as schools, churches, childcare centres, university/research clinic settings (unless available in the community) We also excluded trials that were secondary care based as these are specialized services
**Interventions**	Behavioral interventions focusing on weight loss Interventions may be delivered to children and/or parents via face‐to‐face contact, telephone, print materials, or technology (e.g., computer‐based, text messages), and can be delivered by numerous potential interventionists, including but not limited to physicians, nurses, exercise specialists, dietitians, nutritionists, and behavioral health specialists	Complementary and alternative treatments Surgical and pharmacological treatment Dietary supplements intended for weight loss Exercise or sedentary behavior only interventions
**Comparisons**	No treatment (e.g., wait‐list control, usual care) Attention control (e.g., similar format and intensity to intervention but different content area) Minimal intervention comparable to usual care (including the use of generic printed/electronic communications)	Active comparators without a control (as defined in the inclusion criteria)
**Outcomes**	zBMI and BMI	
**Timing of outcome assessment**	≥ 6 months after start of intervention or baseline assessment (if the intervention start cannot be determined)	

As usual care varies by country, consideration had to be given to the specific nature of which trials would be eligible for inclusion. A pragmatic decision was taken to adopt a definition of usual care being the care that is typically offered in a specific country as stated in the study. Trials were included if they had at least a 6‐month outcome assessment to include as much data as possible. Parent‐only interventions were eligible because evidence from a systematic review indicated children's weight can improve without their attendance, and these interventions may be more cost‐effective [10].

### 2.2 Searches

We conducted a search of the following databases from inception to 06/06/2024: Cochrane Central Register of Controlled Trials, MEDLINE, PubMed, and PsychINFO (Supplementary material 1). An updated search was conducted in PubMed to identify studies published after the initial search up to 05/01/2026. The reference lists of previous reviews [11, 12, 13, 14, 15, 16] and the included trials within these reviews were hand‐searched.

### 2.3 Data Extraction

Results were uploaded to Covidence [17] and duplicates were removed. Two independent reviewers (from among HG, CM, JL, KG, JS, NP, CMc, AD) screened study titles, abstracts, and full texts. Disagreements were discussed and resolved by a third reviewer. All decisions were recorded in Covidence, and reviewers were blinded to the decisions of other reviewers. Data about study characteristics and outcomes were extracted by two independent authors (from among HG, CM, JL, KG, JS, NP, CMc) (Table S1). Twenty‐one authors of included trials were contacted for further information to determine eligibility and for information about the study characteristics and results. Two study authors could not be contacted because the contact details provided in the published papers were out of date. Of the 21 authors contacted, 10 replied with the requested information [17, 18, 19, 20, 21, 22, 23, 24, 25, 26].

### 2.4 Outcomes and Synthesis of Results

The primary outcome was change in zBMI at 12 months. Secondary outcomes were change in BMI at 12 months, change in zBMI at program end, and last follow‐up. Secondary outcomes included quality of life, physical activity, dietary behaviors, reported harms, and progress plus items [27, 28, 29] to examine the equity of interventions. The prespecified subgroup analyses investigated who delivered the intervention, whether the intervention was delivered in or referred to by a primary care setting, country, intensity of the intervention, comparator group, and risk of bias rating. Further details about the subgroup analysis are available in the supplementary file. We used the last available follow‐up data to complete all subgroup analyses.

### 2.5 Statistical Analysis

Meta‐analyses were conducted using Review Manager 5.4 [30]. Random effects models were used as the diversity of intervention components and comparator conditions meant that treatment effects were expected to differ. A pooled mean difference for each analysis was calculated and *I* ^2^ were reported to quantify heterogeneity and Tau^2^ for between study variances. Funnel plots were generated to evaluate small study effects. If there were more than two intervention groups, the number of participants in the comparator group was divided by the number of intervention groups and each was analyzed individually.

### 2.6 Risk of Bias

Two authors (from among HG, CM, KG, AD, JL) independently assessed the risk of bias using the Cochrane Risk of Bias Tool v2 (ROB2) [31]. For incomplete outcome data, a high risk of bias was defined as ≥ 30% attrition and a 20% difference of attrition between the groups. 30% was chosen because in behavioral trials there is greater attrition than drug trials, and in previous trials, 50% has been used, but we took a more conservative approach [32, 33]. Disagreements were resolved by discussion or consulting a third author.

### 2.7 Patient and Public Involvement

The initial idea for the study was discussed with patients and the public and they were in favor of the idea to investigate weight management interventions for children, but they were not included in the design or conduct of the study.

## 3 Results

**FIGURE 1:**
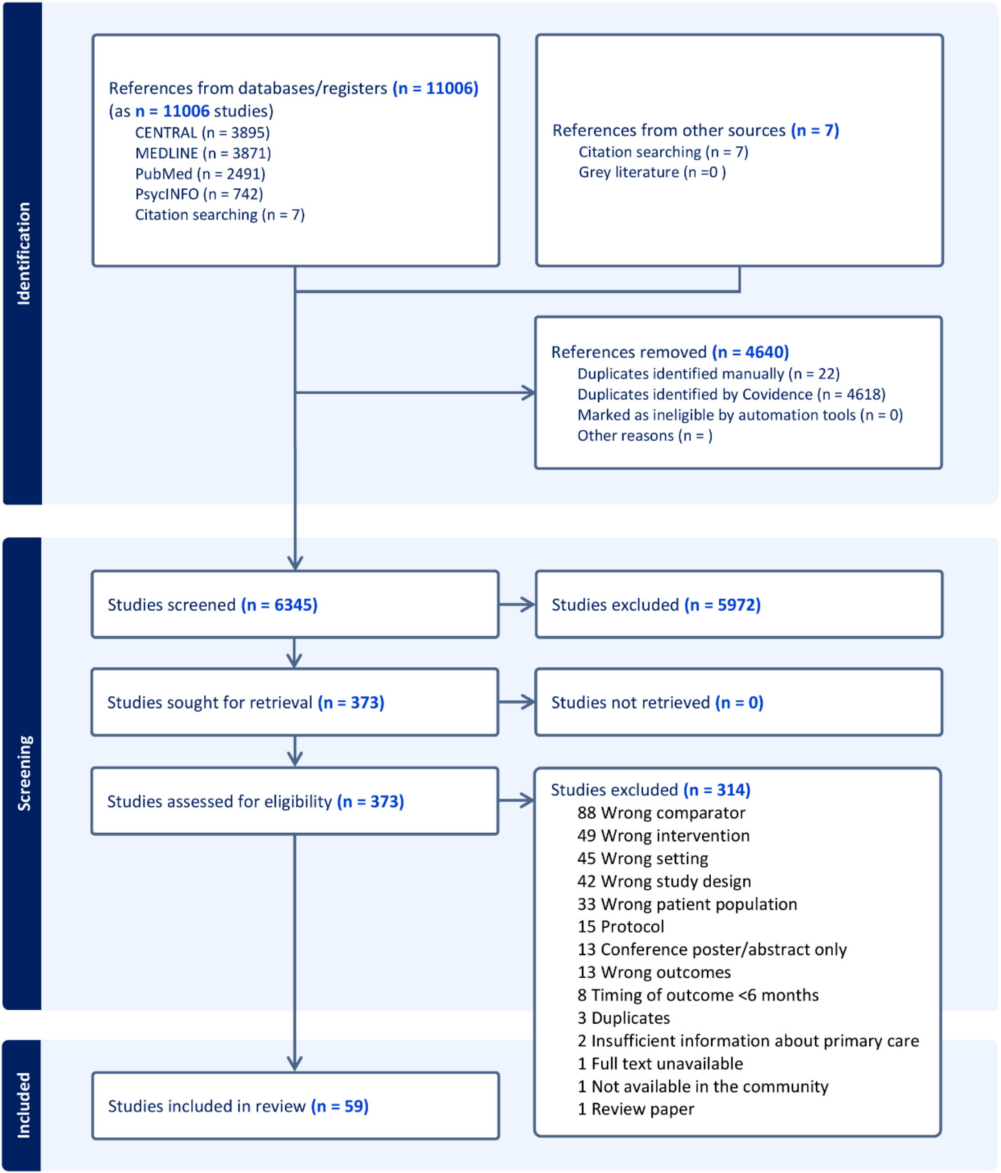
PRISMA flow diagram of screening.

The search identified 6354 unique reports after duplicates were removed (Figure 1). After title and abstract screening, the full texts of 373 reports were assessed for eligibility. From these, 52 (54 publications—two trials each published across two reports [34, 35]) met the inclusion criteria. There were seven additional trials (eight publications—one trial was published across two reports) found through citation searching of systematic reviews and the reference lists of included trials [20, 36, 37, 38, 39, 40, 41]. Therefore, the total number of eligible trials included was 59 (61 publications, 10,454 participants) and of those, six were cluster RCTs [39, 40, 42, 43, 44, 45].

### 3.1 Study Characteristics

The trials were conducted across 14 countries, Brazil [46], New Zealand [35], United States [18, 22, 23, 24, 26, 37, 39, 40, 41, 44, 47, 48, 49, 50, 51, 52, 53, 54, 55, 56, 57, 58, 59, 60, 61, 62, 63, 64, 65], Kuwait [66], UK [19, 27, 38, 67, 68], Canada [69, 70], Italy [36, 71], Sweden [21, 72, 73], Netherlands [45, 74, 75], Denmark [76], Germany [77, 78, 79], Australia [80, 81, 82, 83], Iran [20], Spain [43], and Norway [25]), but most were conducted in the United States (*n* = 29). Sample sizes ranged from *n* = 18 [64] to *n* = 1120 [47]. The duration of interventions ranged from 6 weeks to 2 years (median 6 months). The interventions were delivered in various ways, including face‐to‐face contact [19, 20, 21, 22, 23, 24, 25, 26, 27, 35, 36, 38, 39, 40, 41, 42, 43, 44, 45, 46, 47, 48, 49, 50, 51, 52, 53, 54, 55, 57, 58, 59, 60, 61, 62, 63, 64, 65, 66, 67, 68, 69, 70, 71, 72, 73, 74, 75, 76, 78, 79, 80, 81, 82, 84], phone calls [21, 22, 37, 40, 41, 42, 47, 74, 77], the internet [18, 22, 26, 37, 47, 83], text messaging [18, 22, 47], written documents [22, 37, 55, 59, 60, 71, 81], and via email [26]. However, most interventions were delivered using only face‐to‐face contact or using face‐to‐face contact alongside another method of delivery. Interventions were delivered by a range of different healthcare professionals including, but not limited to, dieticians [19, 21, 35, 36, 42, 43, 44, 47, 50, 54, 56, 65, 66, 67, 69, 70, 73, 75, 79, 83], psychologists/therapists [19, 20, 25, 35, 36, 42, 49, 51, 53, 54, 59, 62, 63, 67, 73, 75, 78, 79], health educators/coaches/counselors/trainers [22, 24, 26, 37, 38, 41, 48, 50, 51, 52, 57, 58, 67, 76, 77], physiotherapists [25, 48, 75], nutritionists [25, 46, 48, 49, 83, 84], pediatricians [25, 37, 40, 41, 43, 48, 49, 55, 63, 71, 73, 78, 82], physicians/GPs [39, 40, 44, 47, 48, 53, 66, 80, 81], research staff (e.g., psychology graduates [27, 50, 60, 62, 64, 70, 85]), and social workers [50, 65, 75]. The average age of participants ranged from 4.1 to 16.5 years (median 9.9 years). The average BMI of participants at baseline was 26.3 (SD = 43.9) kg/m ^2^ (*n* = 34 trials). The average zBMI of participants was 2.4 (0.6) (*n* = 37 trials). Twenty‐six trials reported zBMI change at 12 months (primary outcome) and 16 trials reported BMI change at 12 months.

### 3.2 Risk of Bias

Five trials were considered low risk of bias [27, 75, 77, 82, 86], 43 had some concerns for risk of bias [18, 19, 20, 21, 22, 23, 24, 25, 26, 35, 36, 37, 38, 39, 40, 41, 42, 45, 46, 49, 50, 51, 52, 53, 55, 57, 58, 59, 60, 61, 63, 64, 65, 66, 69, 70, 72, 73, 78, 81, 84, 87], and 11 were defined as high risk of bias [43, 44, 47, 48, 54, 67, 68, 71, 76, 79, 80]. Trials were often rated as having some concern for risk of bias or a high risk of bias because they did not include sufficient information about whether the randomization sequence was concealed from researchers before participants were enrolled. Additionally, trials often included insufficient detail about whether the outcomes were analyzed in accordance with a pre‐specified analysis plan.

### 3.3 Meta‐Analyses of Included Trials

Of the 59 included trials, 46 reported zBMI data. It was not possible to include 9/45 trials [25, 27, 38, 39, 53, 57, 59, 65, 84] that reported zBMI data in any of the meta‐analyses for reasons detailed in the supplementary material 3.

### 3.4 Primary Outcome

**FIGURE 2:**
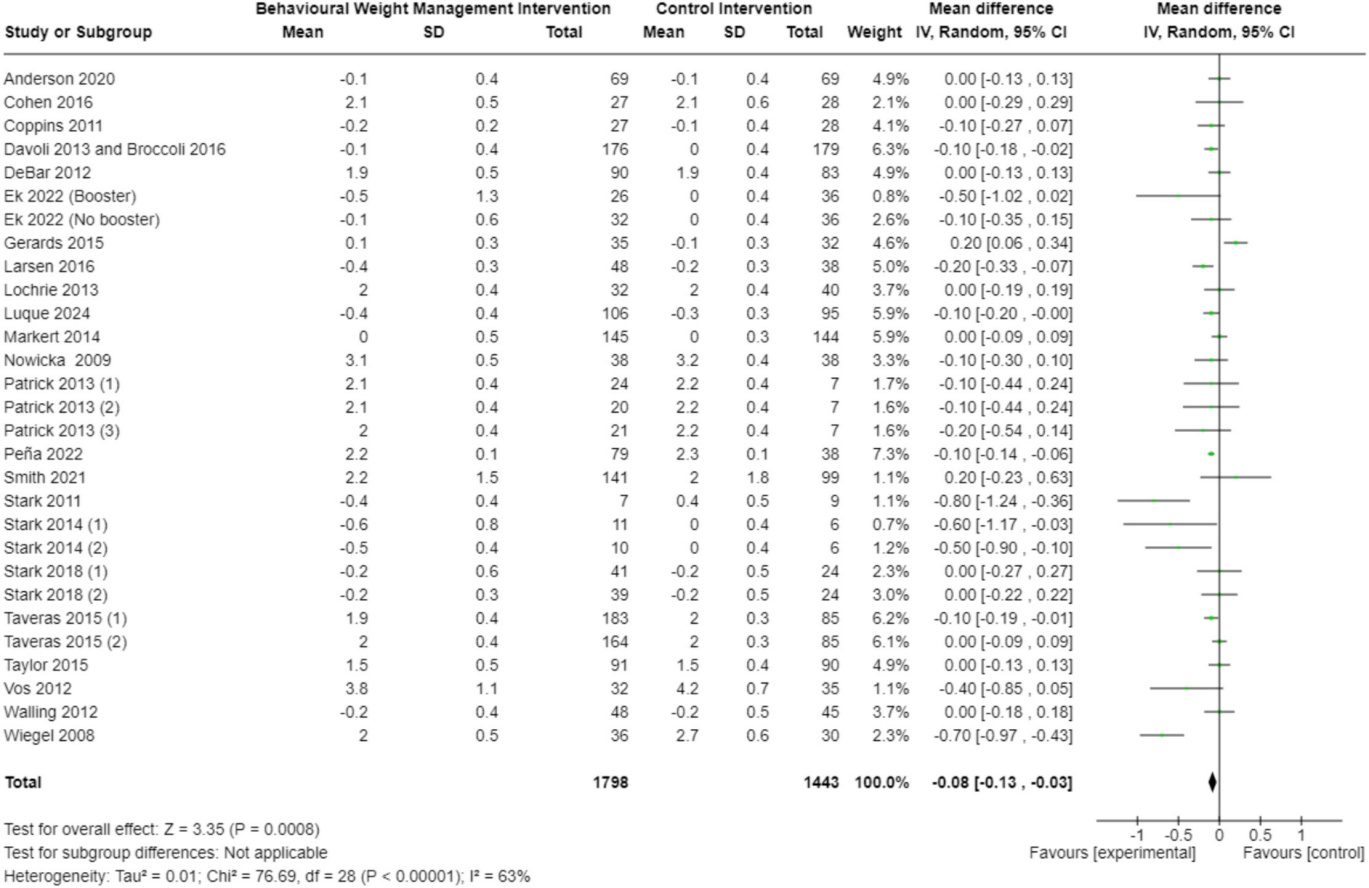
Mean difference in BMI z/SDS at 12 months.

Twenty‐three trials (*n* = 3241) were included in the meta‐analysis for the primary outcome of change in zBMI from baseline to 12 months. The mean difference was −0.08 zBMI (95% CI −0.13 to −0.03, *I* ^*2*^ *=* 63%*,* Tau ^*2*^ = 0.01*, p* < 0.01) (Figure 2), in favor of the intervention group. Inspection of Figure S1 indicates there is unlikely to be evidence of publication bias.

Thirteen of the 36 trials [20, 24, 40, 48, 51, 55, 66, 68, 70, 78, 80, 82, 83] that measured zBMI data did not collect this data at 12 months. One trial [19] did collect zBMI at 12 months but only reported the mean change in zBMI at 12 months for the intervention group, not the comparator.

### 3.5 Secondary Outcomes

#### 3.5.1 Change in BMI at 12 Months

**FIGURE 3:**
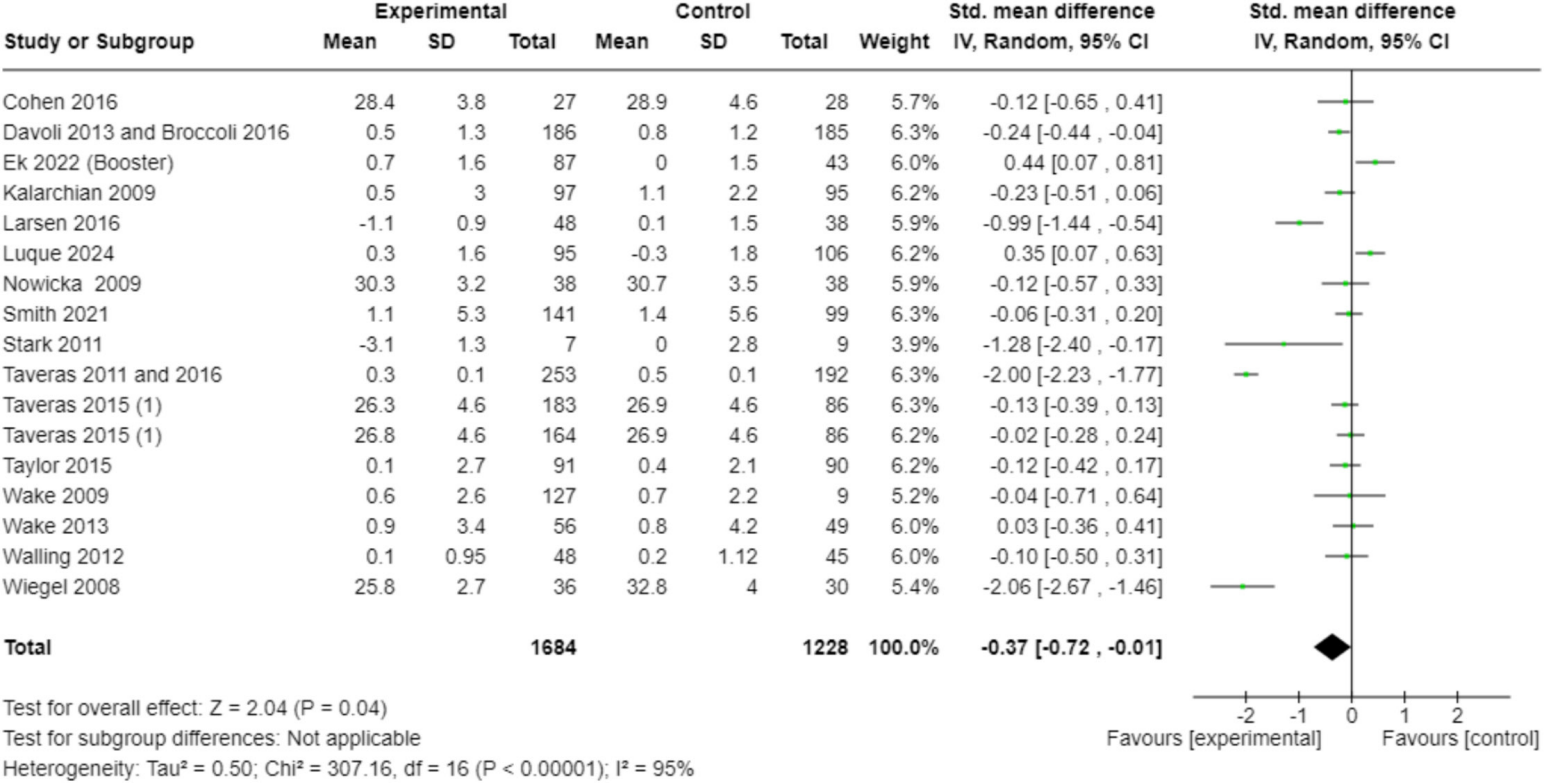
Mean difference in BMI at 12 months.

In total, 16 trials (*n* = 2912) measured BMI at 12 months and there was a mean difference of −0.37 (95% CI −0.72 to −0.01) in favor of the intervention group. There was significant heterogeneity (*I* ^2^ 95%) (Figure 3), and no evidence of publication bias (Figure S2).

#### 3.5.2 Change in ZBMI at Program End

**FIGURE 4:**
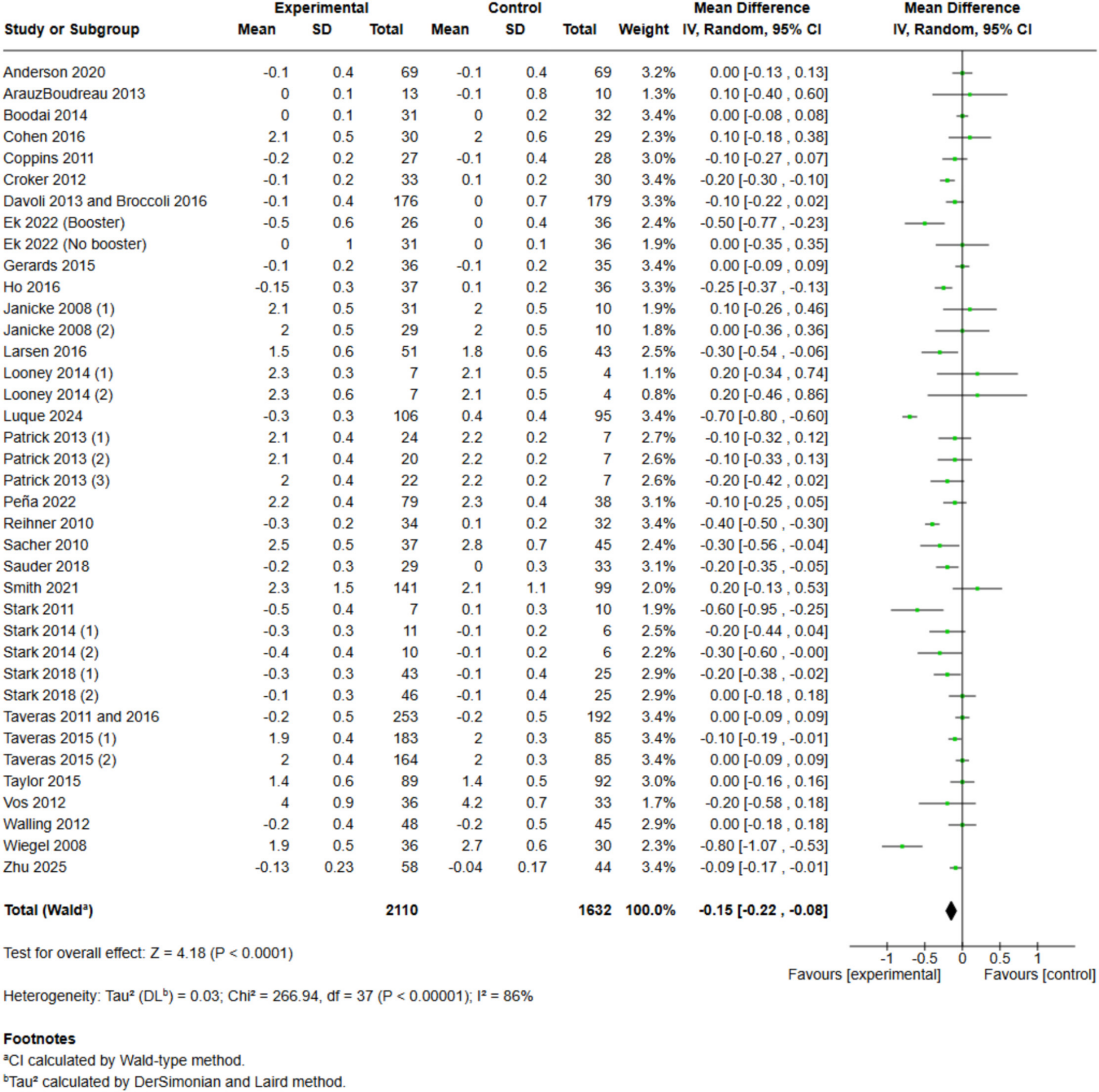
Mean difference in BMI z/SDS at program end.

Thirty trials (*n* = 3742) were included in the analysis of zBMI change from baseline to program end. The mean difference was −0.15 (95% CI −0.22 to −0.08, *I* ^2^ = 86%, Tau^2^ = 0.03, *p* < 0.01), favoring the intervention group (Figure 4). Seven trials [20, 49, 54, 72, 77, 80, 82] did not report zBMI outcomes at program end. Inspection of Figure S3 indicates there is unlikely to be evidence of publication bias.

#### 3.5.3 Change in zBMI at Last Follow‐Up

**FIGURE 5:**
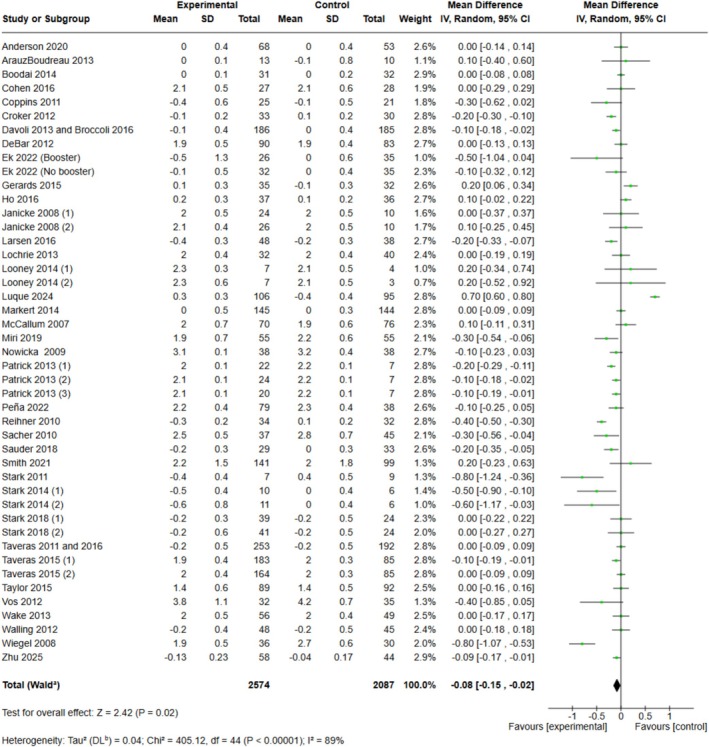
Mean difference in BMI z/SDS at last follow‐up.

Thirty‐seven trials (*n* = 4661) were included in the analysis of zBMI change from baseline to last follow‐up. The mean difference was −0.08 (95% CI −0.15 to −0.02, *I* ^2^ = 89%, Tau^2^ = 0.04, *p* < 0.01), in favor of the intervention group (Figure 5), with no evidence of publication bias (Figure S4).

### 3.6 Subgroup Analyses

We planned to complete subgroup analysis by age group, but it was not possible because there was not enough variation in the ages of children and adolescents within the included studies. We found that interventions where children could be referred to by health professionals in primary care resulted in greater zBMI change (−0.14 [95% CI −0.20 to −0.08] vs. 0.04 [95%CI −0.10 to 0.18]) than those delivered in primary care (Figure S5). There was also a significant difference in the effect of interventions according to the comparator groups (*p* = 0.04). Interventions which were compared to usual care had a smaller change in zBMI (−0.03 [95% CI −0.12 to 0.05]) than waitlist control groups (−0.17 [95% CI −0.27 to −0.06]) and enhanced usual care (−0.31 [95% CI −0.59 to −0.04]) (Figure S6). However, there were small participant numbers in the enhanced care group (*n* = 156).

There were no other statistically significant subgroup differences (Figures S7–S9); however, there appeared to be greater zBMI change in interventions with greater than 12 planned contact points versus less than 12 contact points (−0.13 [95%CI −0.2 to −0.06] vs. 0.00 [95% CI −0.12 to 0.13]) (Figure S10).

### 3.7 Secondary Data Outcomes

We planned to meta‐analyze diet, physical activity, and quality of life, but due to heterogeneity in the outcome measurements used in the trials, these analyses were not feasible. A narrative summary of the findings at 12 months can be found in the supplementary file.

### 3.8 Equity Measures

Ten trials reported no measures of equity, whereas the other trials reported a variety of measures including: parental education, income, country of origin/immigration status, access to health services, marital status and family composition, age, sex, ethnicity, and race. Only 10 studies [21, 27, 35, 36, 40, 44, 47, 50, 52, 65] examined the association of equity measures and the effect on zBMI with mixed results, with no clear conclusions (Supplementary material 5).

## 4 Discussion

### 4.1 Principal Findings

This systematic review of 59 RCTs found that health care professionals delivering or referring young people to behavioral weight management interventions in primary care had small to moderate effects on zBMI/BMI at 12 months but with wide confidence intervals. Findings from baseline to program end resulted in a larger zBMI effect than at 12 months and last follow‐up. While the effects reported in this review are modest, they suggest that behavioral interventions prevent the zBMI/BMI of children living with overweight/obesity from increasing. Pre‐planned subgroup analyses indicated that it may be more effective for primary care health professionals to refer children living with obesity to community‐based behavioral weight management interventions, rather than by direct delivery. Only 10/57 trials examined the association between equity outcomes and the effect of this on zBMI, with mixed results and no clear indication of effects.

### 4.2 Comparison With the Literature

The findings from this systematic review are broadly consistent with the review by O'Conner, which also found that behavioral interventions in health settings were associated with a small reduction in children's BMI after 6–12 months follow‐up [7]. However, the reviews differ in two important ways. The previous review by O'Conner included trials that recruited children from primary care even if the intervention had been delivered in other healthcare settings. Because the specific focus of our review was to provide evidence that could be used directly by commissioners of primary health care services, only interventions that had been delivered in primary care or were referred to by health care professionals within primary care were included. Specifically, interventions conducted in secondary care or hospital settings were excluded as these are specialized services. The O'Conner review included trials with a shorter follow‐up period of 6–12 months and used the follow‐up time point closest to 12 months. In contrast, this review only included trials with follow‐up at 12 months for the primary outcome of zBMI and the secondary analysis of BMI. Making decisions on whether to implement behavioral weight management interventions based on evidence of less than 12 months is problematic because effects will reflect more short‐term and likely favorable changes in zBMI/BMI. Additionally, short(er) follow‐up is less likely to incorporate periods where weight loss might be regained, inflating intervention effectiveness, and potentially increasing cost‐effectiveness values.

A systematic review by Sim et al. published in 2016 that evaluated the effectiveness of brief weight management interventions delivered to children in primary care settings reported these types of interventions to be effective in reducing zBMI [6]. As might be expected from brief interventions, Sims et al.'s review reported a smaller effect for brief interventions than this review (−0.04 versus −0.08 zBMI respectively). Brief interventions may, however, be cheaper to deliver and easier to implement in primary care.

### 4.3 Subgroups Analyses

A further aim of this study was to provide evidence about the contexts (e.g., country) and delivery features of interventions (e.g., which health care professional delivered) to facilitate implementation in primary care settings. Cautious interpretation indicated that referral of children living with overweight/obesity to interventions outside of primary care settings may be more effective than those delivered in primary care. The comparator group also explained some of the variance with statistically significant differences. However, due to small numbers in the different sub‐group categories, we are cautious about this interpretation, particularly for the result of enhanced usual care. The type of health professionals delivering the intervention made no difference to outcomes, and therefore, health coaches could be a cheaper mode of delivery, allowing more children to access support. Indeed, evidence has pointed to health coaches and health coaching as being beneficial in a range of health contexts. For example, a recent systematic review of RCTs tested the effectiveness of health and well‐being coaching compared to any intervention without coaching on chronic disease health outcomes [88]. Meta analyses demonstrated that HWC improved quality of life, self‐efficacy, and depression in adults. Furthermore, the implementation of health coaching is strongly supported by leading health organizations such as the National Health Service in England [89].

Although not statistically significant, there appeared to be greater zBMI change with 12 or more intervention contacts. A previous review by Madigan that investigated the effect of primary care weight management interventions in adults also found that receiving more than 12 contacts was associated with greater weight loss [90]. On average, high‐ and low‐risk trials did not result in differential effects, offering some reassurance that trial quality is unlikely to be driving the effects, although only five trials were graded as low risk of bias.

### 4.4 Strengths and Limitations of the Review

Many trials were included providing the opportunity to conduct sub‐group analyses, to explore heterogeneity about the context in which interventions are most likely to be effective, to optimize implementation. Trials from across different countries and continents were included, increasing the applicability of the findings. Twenty‐one trial authors were contacted for further information and 10 responded, increasing data completeness. Unlike previous systematic reviews, trials that had recruited children of all ages were included [7]. This is important because primary health care has ongoing contact with young children living with overweight/obesity (and their parents) throughout their early years, making this a key period for intervention. Furthermore, it is during the early years of life that dietary and physical activity patterns are formed, highlighting this as the time to intervene. While other reviews have excluded trials defined as low quality, this review included all eligible trials to ensure findings reflected all the available evidence [7]. Our review included a pre‐specified primary outcome in the protocol, which was registered on a publicly available platform, to ensure full transparency in the intended aims and data reporting [91].

The pre‐specified endpoint for the primary outcome was 12 months and 23 trials were included in this analysis. Maintenance of weight loss continues to be a major challenge in healthcare, yet only five trials included follow‐up beyond 12 months, and of these, four included follow‐ups ≥ 24 months. Therefore, no conclusions can be made about the long‐term effects of interventions, and this is an important direction for future research. This question is also a priority for future research because weight relapse and regain are common in those aiming to lose weight [92, 93], and this cycle needs to be reflected in the reporting of effects. A noticeable lack of evidence about the potential longer‐term harms of the interventions to children living with overweight/obesity was identified. Children and young people may be especially vulnerable to experiencing psychological harm (e.g., stress and stigma) from participation in behavioral weight loss interventions [94], and it is imperative that future trials capture this information.

There is debate in the literature as to whether zBMI or BMI is better placed to reflect changes in children's weight following intervention, particularly for children with a BMI above the 97th percentile for age and sex [95]. zBMI was selected here because most trials report findings in this way, maximizing the data available for meta‐analyses. We also analyzed BMI at 12 months, and findings were consistent with those based on zBMI, but there was greater heterogeneity. The authors of this review recognize that zBMI may not fully capture large weight reductions of children with greater excess weight following intervention. If this has been the case, this review may have underestimated intervention effects for zBMI and findings may reflect more conservative estimates rather than inflated ones.

On occasion, it was difficult to determine whether interventions had taken place in primary care or other health settings, such is the fluid nature of healthcare. Different countries adopt different operational definitions of primary care or family health care and at times careful judgment was needed as to whether trials were eligible. Although most authors responded to our requests for additional information or data, some did not. Only five trials (from 57) were defined as having a low risk of bias, with the remainder classified as having some concerns or having a high risk of bias. Of note, 41/59 trials had some concerns for risk of bias, simply because trial authors had not provided sufficient information for review authors to be certain that a criterion had been met. This was particularly the case for whether the randomization sequence had been concealed to researchers prior to participant enrolment and whether outcomes had been analyzed in accordance with a pre‐specified analysis plan. Only three trials had recruited children under 5 years; therefore, findings may be less relevant to very young children. The testing of interventions for this younger age group is certainly an important avenue for future research, not least because overweight tracks across the life course and is associated with later morbidity and mortality [1].

### 4.5 Implications and Future Research

Weight management is known to reduce the risk of a range of physical and psychological health outcomes in children, highlighting the importance of finding effective interventions that can be delivered at scale [96, 97]. It is important to know what findings mean or translate for health improvement. In the case of zBMI, this is difficult to pinpoint, but general estimates have been proposed. Some expert panel groups have proposed that a zBMI reduction of 0.20 should be considered a clinically significant improvement which is typically consistent with a 5% reduction in weight [77, 98]. Similarly, some studies have reported a greater likelihood of meaningful changes in cardiometabolic risk factors with zBMI reductions of 0.15 and 0.125 to 0.50 [99, 100, 101], consistent with findings for program end in this review (zBMI = 0.15). That said, some researchers have proposed a much higher cutoff of zBMI 0.7 to 1.0 [102] and other researchers have not found data to support any particular zBMI threshold [103].

Nevertheless, results should also be interpreted in the context that even a small reduction in weight could be important for children's health and any amount of weight loss that is maintained may reduce the risk of mortality [104]. There are ~427 million children in the world living with overweight or obesity [1] and simply interrupting or slowing down the increase in the zBMI/BMI of even a fraction of the children living with obesity is likely to be important.

In addition to positive health outcomes, the finding that interventions referred to community settings achieved greater zBMI reductions (−0.14 [95% CI −0.21 to −0.07]) than those delivered within primary care (0.04 [95% CI −0.10 to 0.18]) could have economic advantages. This is because referring families to community‐based interventions (e.g., HENRY—weight management program focusing on behavior change strategies, parenting skills and improved knowledge about food and activity for under 5 s and the whole family) [105], rather than delivering in‐house interventions within primary care, could reduce service delivery costs and relieve pressure on healthcare systems.

The broader landscape for intervening to reduce weight in both children and adults has changed in the past 2 years, with the availability of injectable weight loss drugs such as semaglutide. Pharmacological interventions are increasingly being used by doctors to treat obesity in children, but they must be prescribed alongside “wrap‐around care” to support changes in individuals' eating and physical activity behaviors. Although pharmacological interventions were excluded from this review, findings suggest that behavioral weight management interventions can be a useful adjunct (“wrap‐around care”) to support the increasing use of injectable weight loss drugs to treat obesity in children.

## 5 Conclusion

Weight management interventions that are delivered by healthcare professionals or referred to within primary care settings have a small, but important, effect on reducing weight in children living with overweight/obesity. It may be more effective for health professionals to refer children living with obesity to programs outside of primary care rather than to deliver them within this setting.

## Funding

This work was supported by the National Institute for Health and Care Research, 10.13039/501100000272, NIHR300026.

## Conflicts of Interest

ad is supported by a National Institute for Health Research (NIHR) Research Professorship award. CM is supported by a NIHR Advanced Research Fellowship. The views expressed are those of the authors and not necessarily those of the NHS, the NIHR, or the Department of Health and Social Care.

## Supporting information


**Table S1:** Table of characteristics.

## Data Availability

Template data collection forms, extracted data from included studies, and data used for analysis are available from the authors upon request.
