# Supplementary material for: A Systematic Review and Meta‐Analysis of Randomized Controlled Trials of Behavioral Interventions for the Management of Overweight and Obesity in Children That Are Delivered or Referred to by Health Providers in Primary Care
Source: Obes Rev. 2026 Mar 10;27(8):e70119. doi: 10.1111/obr.70119 (PMC13371794; doi:10.1111/obr.70119)
Supplement: Supplementary file 1 — Table S1: Table of characteristics. [file OBR-27-e70119-s001.docx]

**A systematic review and meta-analysis of randomised controlled trials of behavioural interventions for the management of overweight and obesity in children that are delivered or referred to by health providers in primary care**

Henrietta E Graham^1*^ Systematic Reviewer https://orcid.org/0000-0002-0759-7992

Claire D Madigan^2*^ Senior Lecturer/ NIHR Advanced Research Fellow https://orcid.org/0000-0002-6782-0017

Kajal Gokal^2^ Senior Research Fellow https://orcid.org/ 0000-0002-2020-1876

Jessica F Large^2^ PhD Candidate https://orcid.org/0000-0002-5092-7088

James Sanders^2^ Senior Research Associate https://orcid.org/0000-0003-2103-0631

Chris J McLeod^2^ Visiting Fellow in Behavioural Science, Public Health and EDI

Natalie Pearson^1^ Senior Lecturer

Amanda J Daley^2^ Professor of Behavioural Medicine <https://orcid.org/0000-0002-4866-8726>

*Joint first authors

^1^ The Specialist Unit for Review Evidence (SURE), Cardiff University

^2^ Centre for Lifestyle Medicine and Behaviour (CLiMB), ^1^The School of Sport, Exercise and Health Sciences, Loughborough University, UK

**Supplementary material 1: Search strategies**

Medline in Ebsco

1. TI obes* or overweight
2. AB obes* or overweight
3. (MH "Overweight+")
4. (MH "Obesity+")
5. 1 or 2 or 3 or 4
6. TI child* or infant* or infancy or toddler* or baby or babies or adolescent* or teenage* or “young people” or “young person” or “school child*” or pediatr* or paediatr* or boy* or girl* or “pre-school*” or kindergar* or “elementary school*” or “primary school*” or youth*
7. AB child* or infant* or infancy or toddler* or baby or babies or adolescent* or teenage* or “young people” or “young person” or “school child*” or pediatr* or paediatr* or boy* or girl* or “pre-school*” or kindergar* or “elementary school*” or “primary school*” or youth*
8. (MH "Child+")
9. (MH "Infant+")
10. (MM "Adolescent")
11. S6 OR S7 OR S8 OR S9 OR S10
12. TI “weight reduction program*” or “behavio* therap*” or “cognitive therap*” or “counsel*” or “directive counsl*” or “self-help group*” or “health education” or “fat-restricted diet” or “calor* restrict*” or “diet* counsel*” or “diet* education” or “nutrition* counsel*” or “nutrition* education” or “diet therap*” or “nutrion* intervention*” or “diet modifi*” or “diet therap*” or “diet intervention*” or “diet strateg*” or “diet* club*” or “diet* organi?ation*” or “slim* club*” or “slim* organi?ation*” or “weight reduc* diet*” or exercise or “exercise therap*” or “motor activity” or “physical fitness” or “physical activity” or “exercise therap*” or “exercise program*” or “exercise intervention*” or “lifestyle modifi*” or “lifestyle intervention*” or “weight loss intervention*” or “weight loss program*” or “weight loss trial*” or “weight reduc* intervention*” or “weight reduc* program*” or “weight reduc* trial*” or “weight management intervention*” or “weight loss program*” or “weight loss trial*” or “weight control intervention*” or “weight control program*” or “weight control trial*” or “weight loss maintenance intervention*” or “weight loss maintenance program*” or “weight loss trial*”
13. AB “weight reduction program*” or “behavio* therap*” or “cognitive therap*” or “counsel*” or “directive counsl*” or “self-help group*” or “health education” or “fat-restricted diet” or “calor* restrict*” or “diet* counsel*” or “diet* education” or “nutrition* counsel*” or “nutrition* education” or “diet therap*” or “nutrion* intervention*” or “diet modifi*” or “diet therap*” or “diet intervention*” or “diet strateg*” or “diet* club*” or “diet* organi?ation*” or “slim* club*” or “slim* organi?ation*” or “weight reduc* diet*” or exercise or “exercise therap*” or “motor activity” or “physical fitness” or “physical activity” or “exercise therap*” or “exercise program*” or “exercise intervention*” or “lifestyle modifi*” or “lifestyle intervention*” or “weight loss intervention*” or “weight loss program*” or “weight loss trial*” or “weight reduc* intervention*” or “weight reduc* program*” or “weight reduc* trial*” or “weight management intervention*” or “weight loss program*” or “weight loss trial*” or “weight control intervention*” or “weight control program*” or “weight control trial*” or “weight loss maintenance intervention*” or “weight loss maintenance program*” or “weight loss trial*”
14. (MH "Obesity Management+")
15. 12 or 13 or 14
16. TI randomi?ed or “clinical trial*” or “controlled trial*” or “randomi?ed controlled trial*”
17. AB randomi?ed or “clinical trial*” or “controlled trial*” or “randomi?ed controlled trial*”
18. 16 or 17
19. 5 and 11 and 15 and 18

**Limiters** Population Group: Human

PsychInfo in Ebsco:

**Mesh terms don’t exist for infant, child, adolescent and obesity management**

1. TI obes* or overweight
2. AB obes* or overweight
3. DE “overweight” or DE “obesity”
4. 1 or 2 or 3
5. TI child* or infant* or infancy or toddler* or baby or babies or adolescent* or teenage* or “young people” or “young person” or “school child*” or pediatr* or paediatr* or boy* or girl* or “pre-school*” or kindergar* or “elementary school*” or “secondary school*” or “primary school*” or youth*
6. AB child* or infant* or infancy or toddler* or baby or babies or adolescent* or teenage* or “young people” or “young person” or “school child*” or pediatr* or paediatr* or boy* or girl* or “pre-school*” or kindergar* or “elementary school*” or “secondary school*” or “primary school*” or youth*
7. 5 or 6
8. TI “weight reduction program*” or “behavio* therap*” or “cognitive therap*” or “counsel*” or “directive counsl*” or “self-help group*” or “health education” or “fat-restricted diet” or “calor* restrict*” or “diet* counsel*” or “diet* education” or “nutrition* counsel*” or “nutrition* education” or “diet therap*” or “nutrion* intervention*” or “diet modifi*” or “diet therap*” or “diet intervention*” or “diet strateg*” or “diet* club*” or “diet* organi?ation*” or “slim* club*” or “slim* organi?ation*” or “weight reduc* diet*” or exercise or “exercise therap*” or “motor activity” or “physical fitness” or “physical activity” or “exercise therap*” or “exercise program*” or “exercise intervention*” or “lifestyle modifi*” or “lifestyle intervention*” or “weight loss intervention*” or “weight loss program*” or “weight loss trial*” or “weight reduc* intervention*” or “weight reduc* program*” or “weight reduc* trial*” or “weight management intervention*” or “weight loss program*” or “weight loss trial*” or “weight control intervention*” or “weight control program*” or “weight control trial*” or “weight loss maintenance intervention*” or “weight loss maintenance program*” or “weight loss trial*”
9. AB “weight reduction program*” or “behavio* therap*” or “cognitive therap*” or “counsel*” or “directive counsl*” or “self-help group*” or “health education” or “fat-restricted diet” or “calor* restrict*” or “diet* counsel*” or “diet* education” or “nutrition* counsel*” or “nutrition* education” or “diet therap*” or “nutrion* intervention*” or “diet modifi*” or “diet therap*” or “diet intervention*” or “diet strateg*” or “diet* club*” or “diet* organi?ation*” or “slim* club*” or “slim* organi?ation*” or “weight reduc* diet*” or exercise or “exercise therap*” or “motor activity” or “physical fitness” or “physical activity” or “exercise therap*” or “exercise program*” or “exercise intervention*” or “lifestyle modifi*” or “lifestyle intervention*” or “weight loss intervention*” or “weight loss program*” or “weight loss trial*” or “weight reduc* intervention*” or “weight reduc* program*” or “weight reduc* trial*” or “weight management intervention*” or “weight loss program*” or “weight loss trial*” or “weight control intervention*” or “weight control program*” or “weight control trial*” or “weight loss maintenance intervention*” or “weight loss maintenance program*” or “weight loss trial*”
10. 8 or 9
11. TI randomi?ed or “clinical trial*” or “controlled trial*” or “randomi?ed controlled trial*”
12. AB randomi?ed or “clinical trial*” or “controlled trial*” or “randomi?ed controlled trial*”
13. 11 or 12
14. 4 and 7 and 10 and 13

Age Groups: Childhood (birth-12 yrs), Neonatal (birth-1 mo), Infancy (2-23 mo), Preschool Age (2-5 yrs), School Age (6-12 yrs), Adolescence (13-17 yrs); Population Group: Human

PubMed

(((("obes*"[Title/Abstract] OR “obesity” [Mesh] OR "overweight"[Title/Abstract] OR “overweight” [Mesh]) ) AND (("child*"[Title/Abstract] OR “child” [Mesh] OR “infant*” [Title/Abstract] OR “infancy” [Title/Abstract] OR “toddler*” [Title/Abstract] OR “baby” [Title/Abstract] OR “babies” [Title/Abstract] OR “infant” [Mesh] OR "adolescent*" [Title/Abstract] OR "teenage*"[Title/Abstract] OR “adolescent” [Mesh] OR "young people"[Title/Abstract] OR "young person"[Title/Abstract] OR "school child*"[Title/Abstract] OR "pediatr*"[Title/Abstract] OR "paediatr*"[Title/Abstract] OR "boy*"[Title/Abstract] OR "girl*"[Title/Abstract] OR “pre-school*” [Title/Abstract] OR “kindergar*” [Title/Abstract] OR “elementary school*” [Title/Abstract] OR “primary school*” [Title/Abstract] OR “secondary school*” [Title/Abstract] OR "youth*"[Title/Abstract]))) AND ("weight reduction program*"[Title/Abstract] OR "cognitive therap*"[Title/Abstract] OR "counsel*"[Title/Abstract] OR "self help group*"[Title/Abstract] OR "health education"[Title/Abstract] OR "fat-restricted diet"[Title/Abstract] OR "diet counsel*"[Title/Abstract] OR "nutrition counsel*"[Title/Abstract] OR "diet therap*"[Title/Abstract] OR "diet modifi*"[Title/Abstract] OR "diet intervention*"[Title/Abstract] OR "diet strateg*"[Title/Abstract] OR "diet club*"[Title/Abstract] OR "slim club*"[Title/Abstract] OR "exercise"[Title/Abstract] OR "exercise therap*"[Title/Abstract] OR "motor activit*"[Title/Abstract] OR "physical fitness"[Title/Abstract] OR "physical activit*"[Title/Abstract] OR "exercise program*"[Title/Abstract] OR "exercise intervention*"[Title/Abstract] OR "lifestyle modifi*"[Title/Abstract] OR "lifestyle intervention*"[Title/Abstract] OR "weight loss intervention*"[Title/Abstract] OR "weight loss program*"[Title/Abstract] OR "weight loss trial*"[Title/Abstract] OR "weight management intervention*"[Title/Abstract] OR "weight control intervention*"[Title/Abstract] OR "weight control program*"[Title/Abstract] OR "weight control trial*"[Title/Abstract] OR "weight loss maintenance intervention*"[Title/Abstract] OR "weight loss maintenance program*"[Title/Abstract])) AND ("randomi"[Title/Abstract] OR "clinical trial*"[Title/Abstract] OR "controlled trial*"[Title/Abstract])

Cochrane:

#1 obes* or overweight Title Abstract Keyword

#2 MeSH descriptor: [Overweight] explode all trees

#3 MeSH descriptor: [Obesity] explode all trees

#4 #1 or #2 or #3

#5 child* or infant* or infancy or toddler* or baby or babies or adolescent* or teenage* or “young people” or “young person” or “school child*” or pediatr* or paediatr* or boy* or girl* or “pre-school*” or “kindergar*” or “elementary school*” or “primary school*” or “secondary school*” or youth*

#6 MeSH descriptor: [Child] explode all trees

#7 MeSH descriptor: [Infant] explode all trees

#8 MeSH descriptor: [Adolescent] explode all trees

#9 #5 or #6 or #7 or #8

#10 (“weight reduction program*” or “behavio* therap*” or “cognitive therap*” or “counsel*” or “directive counsl*” or “self-help group*” or “health education” or “fat-restricted diet” or “calor* restrict*” or “diet* counsel*” or “diet* education” or “nutrition* counsel*” or “nutrition* education” or “diet therap*” or “nutrion* intervention*” or “diet modifi*” or “diet therap*” or “diet intervention*” or “diet strateg*” or “diet* club*” or “diet* organi?ation*” or “slim* club*” or “slim* organi?ation*” or “weight reduc* diet*” or exercise or “exercise therap*” or “motor activit*” or “physical fitness” or “physical activit*” or “exercise therap*” or “exercise program*” or “exercise intervention*” or “lifestyle modifi*” or “lifestyle intervention*” or “weight loss intervention*” or “weight loss program*” or “weight loss trial*” or “weight reduc* intervention*” or “weight reduc* program*” or “weight reduc* trial*” or “weight management intervention*” or “weight loss program*” or “weight loss trial*” or “weight control intervention*” or “weight control program*” or “weight control trial*” or “weight loss maintenance intervention*” or “weight loss maintenance program*” or “weight loss trial*”):ti,ab,kw

#11 MeSH descriptor: [Obesity Management] explode all trees

#12 #10 or #11

#13 (randomi?ed or “clinical trial*” or “controlled trial*” or “randomi?ed controlled trial*”):ti,ab,kw

#14 #4 and #9 and #12 and #13

**Supplementary material 2: Subgroup analysis**

1. Delivered in primary care – studies were divided to those that were delivered in primary care or those that could be referred to by primary care.
2. Professional delivering intervention—studies were divided into those that were delivered by GPs, paediatricians, nurses, dieticians or a combination of these and those delivered by health coaches.
3. Country—We explored the effectiveness of interventions by country. There were only two countries with four or more studies (US and UK) and therefore we grouped the other studies into European countries and Australasian countries.
4. Intensity of interventions—we categorised intervention groups according to whether ≤11 or ≥12 contacts.
5. Risk of bias rating—Studies were classified as being at low, unclear, and high risk of bias. Risk of bias was explored as a potential influence on the results.
6. Comparator – We explored whether the comparator influenced differences in BMIz/ SDS change by usual care, wait list control and more intensive comparators.

**Supplementary material 3: Reasons studies that reported zBMI were not included in the meta-analyses**

Two studies reported the mean zBMI change within their manuscripts, but it was not clear whether the mean change reported was from baseline to 6 months or from baseline to 12 months (Byrant & Christie). One study did not report the number of participants included in their analysis of mean zBMI change (O’Connor). One study reported mean zBMI change from baseline to 6 months for the intervention group but from baseline to 12 months for the comparator group (Skjåkødegård 2022). Two studies only displayed the mean zBMI change in graphs (Wylie-Rosett & Raynor). One study only reported zBMI change with interquartile ranges (Kumar). One study did not report 95% CIs or SDs (Mellin) and one study only reported mean change in zBMI for the intervention group (Raynor). All authors were contacted regarding the issues above, but further information/data was not provided.

**Supplementary material 4: Narrative summary of secondary outcomes at 12 months**

Dietary outcomes

There were 21 studies that reported they measured dietary outcomes.

Anderson

Brandao

Cohen

Coppins

Davoli

DeBar

Gerards

Janicke

Looney

O’Connor

Reihner

Saelens

Stark 2014

Stark 2018

Wake

Newsome

Chen

Miri

Patrick

Raynor

Zhu

Of those only seven reported differences at 12 months in dietary outcomes (Anderson, Gerrads, Stark 2014, Stark 2018, Raynor 2012). Anderson measured fruit and vegetable servings, Gerards fruit servings per week, Janicke and Stark 2014, Stark 2018, Raynor and Zhu 2025 measured, energy intake (kcals) per day. Brandao measured diet using a planetary health diet index. Anderson, Gerards, Brandao and Raynor found no significant mean differences between the groups. Whereas Stark 2014 found significant mean differences in favour of the two intervention groups compared to the comparator of -640 (95% CI -932 to -348, LAUNCHJ – HV) and -415 (95% CI -734 to -07, LAUNCH- Clinic). Stark 2018 also found significant differences in favour of the intervention group (mean difference of 284 (95% CI 160 to 408). Zhu found significant differences in favour of the intervention group (p=0.01). Brandao found no

Quality of Life

Only five studies reported quality of life at 12 months and there were no reported significant differences between the groups.

Anderson

DeBar

Vos

Kalarchian

Zhu

Physical activity

Only eight studies provided data about differences in physical activity at 12 months.

Anderson

Gerards

Stark 2014

Stark 2018

Taylor

Sacher

Skjakodegard

Zhu

Anderson measured steps per day, Gerrad, Taylor and Stark (2014 and 2018) and Skjakodegard used an accelerometer. Gerard and Taylor calculated minutes of moderate and vigorous physical activity (MVPA) per day, Stark (2014 & 2018) calculated moderate minutes per day and Skjakodegard calculated percentage of time in a day of MVPA. Sacher used an invalidated questionnaire to measure physical activity. Zhu used a questionnaire to measure physical activity. Only the study by Zhu found a significant difference between the groups in favour of the control group (p=0.02).

**Supplementary material 5: Studies that have explored outcomes by equality measures**

Tavera 2011- Found significant reductions of BMI in females compared to no significant reductions in males. Significant reductions of BMI in household incomes <$50,000 compared to those with higher incomes. No difference in ethnicity, race, education.

Ek – Fathers from foreign backgrounds was associated with less BMI change but no effect on parental education, income or make up of home (i.e. living with both parents.

Davoli- If mothers' education was <13 years it was associated with less BMI change.

Andersen – If participants were younger there was greater BMI channge and deprivation was associated with less improvements. No difference in sex. NZE participants had greater change in BMI compared to Maori participants.

Epstein 2023 – White race was associated with significantly greater BMI change, but no difference in sex, household income and parent education.

Reniscow 2024 – Black and other youth associated with less BMI change.

Christie – no difference based on sex

Reniscow 2015 – no interaction based on age, gender, income and education on BMI change.

Kalarachian – higher income associated with larger decreases in percentage change in weight.

Wylie-Rosett – females and all ethnicities compared to non-hispanic black participants lost less weight.

**Table S1: Table of characteristics**

| **Study ID**  **Country, n** | **Inclusion criteria** | **Mean age (years) (SD)** | **Female %** | **White ethnicity %** | **Mean BMI/ BMI Z score at baseline (SD)** | **Intervention** | **Deliverer and Training received** | **Comparator** | **Duration and Follow-ups** |
| --- | --- | --- | --- | --- | --- | --- | --- | --- | --- |
| Anderson 2017 & 2020, n=121 | 5-16 yrs, BMI ≥98th percentile or BMI >91st percentile with weight-related comorbidities | 10.34 (3.03) | 50 | 47.4 | 28.5 (5.0)/ 3.02 (0.55) | Group intervention face to face with home-based assessments, healthy lifestyle advice, and weekly activity sessions. | Activity coordinator, dietitian, and psychologist  NR | Usual care: low-intensity healthy lifestyle only intervention with home-based assessments | 12 months  F-up: 12 & 24 months |
| ArauzBoudreau 2013, US, n=41 | Latino children aged 9-12 yrs living with overweight or obesity who received primary care at a single community health centre | 10.3 (1.3) | 61.5 | NR | NR/ 2.1 (0.3) | (1) five weekly Power Up face to face classes about healthy behaviours (nutrition, activity, and stress management) and (2) culturally sensitive coaching to empower families to incorporate learned behaviours and address both family and social barriers to lifestyle changes. | Health educators, physical therapists, nutritionists and a primary care paediatrician  NR | Wait list control | 24 weeks, f-up: 6 months |
| Boodai 2014, Kuwait, n=82 | 10-14 yrs with obesity with at least one parent who expressed a willingness to attend the intervention | 12.4 (1.2) | 49 | NR | NR/ 2.2 (0.3) | 6 group discussion sessions face to face (1 hr each), focusing on a reduction in SB, modified version of traffic light diet system, promotion of PA with BCTs delivered to adolescents and parents. | Physician and dietician  Specialist training in nutrition | Usual care | 24 weeks f-up- 6 months |
| Brandão 2025, Brazil, n=120 | 7-12 yrs with overweight or obesity (Z-score ≥ 1.5) | 9.4  (1.57) | 59.2 | 32.5 | 28.0 (4.9)/NR | 6 in-person sessions (first two spaced 15 days apart, the others were monthly) that included educational materials, monthly diet goals aligned to a Brazilian-adapted Planetary Health Diet, portion control utensils, home-based PA support, goal tracking, online exercise content, sports equipment, and motivational messages. | Nutritionists and physical education professionals  NR | Usual care | 4 months, f-up: |
| Byrant 2011, UK, n=70 | 8-16 yrs with a BMI > 98th percentile value and a parent/carer with fluent spoken English | 11.4 (2.0) | 64 | 87 | NR/ 2.99 (0.46) | 4-month motivation-enhancing solution focused intervention embedded within Primary Care Trust Services in Leeds, which could be extended for a further 4-8 months. Weekly individual appointments structured on the Healthy Eating Lifestyle Programme and group physical activity sessions. | Health trainers  NR | Waitlist control | 4 months (option to continue for a further 4-8 months)  f-up: 6 & 12 months |
| Chen 2017, US, n=40 | 13-18 yrs, BMI ≥ 85th percentile | 14.9 (1.67) | 42.5 | NR | 27.8 (3.7)/ 1.5 (0.5) | 3-component intervention, wearable sensor (Fit-bit Flex), 8 online educational modules for 3 months and received tailored, biweekly text messages for 3 months. | Delivered remotely (no contact with deliverer)  NR | Self-monitored PA, SB and food intake and accessed an online program that consisted of 8 modules related to general adolescent health issues. | 3 months  f-up: 3 & 6 months |
| Christie 2017, UK, n=174 | 12-19 yrs, BMI >95th centile for age and sex | 15 (NR) | 63 | 48 | 32 (NR)/ 2.8 (NR) | 12-session family-based face to face weight-management programme that included motivational interviewing and solution-focused approaches to increase engagement and concordance. | Psychology graduates  5-day training programme on obesity and good clinical practice | 40–60 mins standardised educational session incorporating Department of Health guidance on eating behaviours, healthy eating and activity, delivered by a primary care nurse in the participant’s general practice within 3 months of recruitment. | 26 weeks  f-up: 6 & 12 months |
| Cohen 2016, Canada, n=60 | 9-12 yrs with no known illness, living with overweight or obesity | 11.1 (1.2) | 53.3 | 96.7 | 27.5 (4.0)/ 2.7 (0.6) | Family centred lifestyle intervention based on Canadian dietary and PA guidelines, included six monthly sessions focused on behavioural counselling and motivational interviewing. | Dietician  NR | Waitlist control | 6 months,  f-up:  3, 6, 9 & 12 months |
| Coppins 2011, UK, n=65 | 6-14 yrs with a BMI > 91st percentile | 10.4 (NR) | 66.5 | NR | 27.5/ (NR)/ 2.8 (NR) | 2 workshops face to face) (focused on healthy eating, PA, reducing SB, behaviour change and psychological wellbeing), 1 week apart and 2 PA sessions of 1hr/week during term time for the 1-year intervention. | Dietician, PA health promotion officer, an educational or clinical psychologist and 2–3 PA instructors  NR | Waitlist control | 12 months  f-up: 6, 12, 18 & 24 months |
| Croker 2012, UK, n= 72 | 8-12 yrs living with overweight or obesity, had at least one parent or guardian willing to participate, sufficient command of English | 10.3 (1.6) | 69.4 | 56.9 | 30.6 (5.3)/ 3.2 (0.6) | Behavioural weight control programme with 15 group sessions face to face (8-10 families per group, 10 weekly, 3 fortnightly, 2 monthly sessions) that aimed to reduce fat and energy intake, increase PA and change parent-child interactions. | Parents’ groups: psychologist, family therapist or  experienced dietitian  Children’s groups: dietitian  NR | Waitlist control | 26 week f-up: 6 months |
| Davoli 2013 & Broccoli 2016, Italy, n=372 | 4-7 yrs with overweight | 6.6 (1.1) | 62 | NR | 18.2 (NR)/ 1.35 (NR) | MI based intervention consisting of 5 individual face to face meetings based on the transtheoretical model of addiction and behaviour change. | Parents’ groups: psychologist, family therapist or  experienced dietitian  Children’s groups: dietitian.  NR | Usual care: booklet with the main information on obesity prevention and usual care currently offered by paediatricians to children living with overweight | 12 months intervention and f-up. |
| DeBar 2012, USA, n=208 | Females, 12-17 yrs, age- and gender-adjusted BMI>=90th percentile | 14.1 (1.5) | 100 | 72.1 | 31.9 (4.7)/ NR | Multi-component intervention consisting of sixteen 90-min group meetings about diet and PA. 12 group sessions for parents to help parents support their daughters and reduce potential barriers to successes. | Master’s level nutritionists and health educators, doctoral level clinical psychologists and paediatricians.  Training in motivational enhancement techniques for health behaviour change. | Usual care: received materials, including outlines of evidence-based approaches to weight management for youth and adults, a parents’ guide to help adolescents make healthy lifestyle changes, local resources. Met with their PCPs at the study onset to encourage healthy lifestyle changes. | 5 months, f-up- 6 & 12 months |
| Epstein 2023, USA, n= 452 families | Families with a child aged 6-12 years with overweight or obesity (>85th percentile BMI), a parent with overweight or obesity  (BMI >25) | 9.8 (2.2) | 44 | 56 | 26.6 (5.4)/ NR | Standard treatment and a family-based treatment including Traffic Light Eating and Activity Plans, parenting and behavioural techniques, and facilitation of support in family and peer environments. Weigh-ins, review of eating and activity self-monitoring in habit books, review of weight change and problem solving and goal setting. | Coaches with  experience of working with families. In all cases but 1, individuals  either had master’s degrees in psychology, counselling, or social  work or were master’s degree–level registered dietitians.    NR | Usual care | 24 months, f-up - 6, 12, 18, and 24 months |
| Ek 2019, Sweden, n=174 | 4-6 yrs, diagnosed with obesity, had no other chronic diseases or developmental problems likely to influence child weight and height | 5.3 (0.8) | 56.3 | NR | 21.4 (1.8)/ 3.0 (0.6) | 12-weekly 90-min sessions with parents-only: provided support to parents via positive parenting practices to improve parent-child communication.  Booster: 30-min phone calls every 4 to 6 wks for 9 months to encourage parents to maintain healthy habits and provide support for challenges. No booster: did not receive the phone calls. | Dietician  NR | Usual care: Parents and children attended treatment together with a paediatrician and paediatric nurse that focused on healthy food choices and active lifestyle habits. Some children were referred to dieticians and/or physiotherapists. | 12 months (booster), 3 months (no booster). F-up- 3,6 & 12 months |
| Gerards 2015, Netherlands, n=86 | Parent-child triads and was able to communicate in Dutch and had a child 4-8 yrs with overweight or obesity | 7.2 (1.4) | 56 | NR | 20.5 (3.0)/ 1.8 (0.8) | Parents received a 14-wk intervention consisting of ten 90-min group sessions and 4 individual telephone sessions of active skills training methods based on SR principles (nutrition, PA and positive parenting strategies). Individual telephone sessions provided for parents. | Lifestyle Triple P facilitators ('health professionals').  3-day Triple P training course and an additional Lifestyle Triple P Day. | Usual care: 2 brochures as well as a short knowledge quiz via the Internet including tailored advice and suggestions for active exercises at home. | 14 weeks, f-up 4 & 12 months |
| Ho 2016, Canada, n=99 | Age 8 years to  16 years and had a BMI ≥85th percentile for age and gender | 11 (2.2) | 55 | NR | 29.1 (5.6)/ 2.7 (0.4) | 1-hour session of standard nutrition counselling from a registered dietitian.  An additional 10 to 15 minutes counselling  on how to use a calibrated dinner plate and breakfast  cereal bowl for the child and adults in the family as a means of dietary portion control. | Dietician + research coordinator  NR | 1-hour session of standard nutrition counselling from a registered dietitian. | 6 months, f-up- 6 months |
| Janicke 2008, US, n=93 | 8-14 yrs, with a BMI > 85th percentile for age and sex, physician approval to participate in the study | 11.1 (NR) | 60.6 | 76.1 | NR/ 2.1 (NR) | Two interventions: a behavioural FB intervention and a behavioural PO intervention. Weekly 90 min group sessions for 8 wks, then biweekly for 8 wks. Monitor food intake so f to set daily dietary goals at the end of each session. **FB intervention**: parent and child dyads participated in simultaneous groups sessions. **PO intervention**: only parent attended and sessions were similar to parent sessions of the FB intervention with emphasis placed on teaching parents to work with their children to set goals. | Family and Consumer Sciences agents in collaboration with a postdoctoral psychologist, and undergraduate students in clinical psychology  2 full days of training and 6 hours of booster training midway through the intervention | Waitlist control | 16 weeks, 4 & 10 months |
| Kalrachian 2009, US, n=192 | 8-12 yrs, BMI of 97th percentile, adult willing to participate in the program with the child | 10.2 (1.2) | 56.8 | 73.4 | 32.1 (4.95)/ NR | Family-based intervention, group-based sessions for children and adults separately. Participants were provided with a modified version of the Stoplight Eating Plan and taught behavioural strategies to increase PA and to decrease SBs. | Lifestyle coaches  NR | Usual care: offered 2 nutrition consultation sessions to develop an individual nutrition plan based on the Stoplight Eating Plan and offered the intervention after completion of the 18-month assessment. | 24 weeks, f-up 6 & 12 months |
| Kumar 2018, US, n=22 | 14-17 yrs, BMI ≥ 95th percentile for age and gender | Median: 16.4 | 45.5 | 86.4 | 33.9 (NR)/ 2.5 (NR) | Face-to-face  Mindful eating program over four 90-minute sessions. | Physician and mind-body therapist  NR | Usual care: three 90 min sessions of dietary counselling by a registered paediatric dietician at baseline, 12 wks and 24 wks. | 10 weeks, f-up- 12 and 24 wks |
| Larsen 2016, Denmark, n=115 | Fifth grade primary school children in municipality of Odense living with overweight or obesity | 12.0 (0.4) | 55.7 | NR | NR/ 1.94 (0.49) | Six-week day-camp focusing on increased PA, and healthy diet, followed by a subsequent one-year family-based intervention including four joint meetings that involved discussing and sharing experiences related to a chosen topic. After the second meeting an “activity day” was arranged for the children. | Day camps: 'Trained instructors', family-based intervention: trained school nurses and instructors from the day-camp intervention.  NR | Weekly exercise session (2 hrs) for 6 weeks, as well as a single health and lifestyle educational session for the parents, delivered by a dietician and a physical activity specialist. | 6 weeks, f-up 6 & 52 weeks |
| Lochrie 2013, USA, n=130 | 8-11 yrs, age- and sex-adjusted BMI >=85th percentile, did not have impaired glucose tolerance, DM2, MS, hypertension, or significant learning problems | 9.9 (1.1) | 63 | 49 | NR/ 2.2 (0.4) | 8 weekly sessions, followed by 4 bimonthly sessions, and then 2 monthly sessions (60-90 mins) that utilised The Committed to Kids Pediatric Weight Management Program and covered topics in nutrition, behaviour modifications, psychological interventions, exercise topics and medical issues related to obesity. | Psychologists and dieticians  NR | One 1-hr group session (2-9 families) led by a registered dietitian who provided general recommendations to the families regarding nutrition and PA goals (similar to the info given to the IG) and a list of resources in the community for weight management. | 24 weeks, 8.1 & 14.3 months |
| Looney 2014, US, n=22 | 4-to 10 yrs with overweight or obesity | 8.0 (1.8) | 68 | NR | NR/ 2.34 (0.46) | Two conditions: newsletter and growth monitoring (N + GM) & newsletter, growth monitoring and family-based behavioural counselling (N + GM + BC). **N + GM**: received usual care, monthly educational newsletters about nutrition and PA and monitored and received monthly feedback about their child’s growth over 6 months. **N+GM+BC**: Received everything the N + GM condition did as well as monthly contact (3, 30-min in person appointments and 3, 20-min phone calls). Verbal feedback about growth of the child. and counselling the caretaker using behavioural strategies to aid with changing 2 dietary leisure time behaviours pf the caretaker or child. | Trained interventionist and paediatrician  NR | Enhanced usual care: in addition to the child’s usual care, they were mailed 6 monthly educational newsletters on nutrition and leisure-time activity topics. | 24 weeks f-up: 6 months |
| Luque 2024, Spain, n=303  (Cluster RCT) | Aged 8-14 years, diagnosed with obesity (BMI greater than the 97^th^ percentile), and attending to regular health checks at primary care centres | 128 (6) months | 45 | NR | 25.7 (2.6)/ 2.61 (0.4) | Multicomponent intervention (diet and physical activity) with monthly interviews with patients and families (up to  11 interviews) and the completion of questionnaires. | Paediatricians and paediatric nurses, and dieticians  NR | Standard care following: Guidelines for Clinical Practice. | 12 months, f-up, 12 months |
| Markert 2014, Germany, n=289 | 4-17 yrs, BMI > 90th percentile | 9.8 (3.0) | 50.5 | NR | 24.2 (3.9)/ 2.0 (0.5) | Computer-aided telephone counselling (20-30 mins each x 14 [two additional phone calls offered]) for one year based on family therapy approaches and solution-focused systemic therapy. | Trained prevention managers  NR | NR | 52 weeks, f-up -12 months* |
| McCallum 2007, Australia, n=163 | All children classified as living with overweight or obesity in the BMI survey, who were not receiving ongoing weight management in a secondary or tertiary care programme | 7.4 (1.6) | 52 | NR | 20.3 (2.0)/ 1.9 (0.5) | GPs used a brief solution-focused approach to set and record appropriate, healthy lifestyle goals with the family, assisted by a personalized ‘Family Folder. | GP  NR | Usual care: notified of their status via letter and were not identified to the GPs at any time. | 12 weeks, f-up - 9 & 15 months (supposed to be 6 & 12 months) |
| Mellin 1987, US, n=66 | NR | 15.6 (NR) | 79 | NR | NR | 14 wkly 90-minute sessions including weigh ins, group interactions to make successive small modifications in diet, exercise, communication and an exercise period. Parents were instructed on strategies for supporting their adolescents weight loss efforts. | Nutritionists/registered dietitians  NR | Waitlist control | 14 weeks, f-up- 3 and 15 months |
| Miri 2019, Iran, n=110 | 13-18 yrs, BMI 85th percentile for age and gender, ability to attend CBT sessions | 14.76 (1.6) | 47.3 | NR | NR/ 2.14 (0.61) | Six (30-45 mins) face-to-face CBT sessions about nutritional recommendations and diet, self-monitoring diet and PA. | Trained therapists  An experienced CBT therapist trained the intervention therapists for over 100 hrs of supervision. | Usual care that focused on lifestyle modification including diet plus exercise. | 6 weeks, f-up- 6 months |
| Nova 2002, Italy, n=186 | 3-12 yrs living with obesity (excess weight (≥20% of ideal body weight) | 8.6 (2.0) | 44 | NR | 22.9 (2.15)/ NR | One brief contact and information leaflet with a specific diet, detailed guidelines regarding PA and active parental commitment, and an alimentary diary with instructions. | Paediatrician  NR | Usual care: leaflets that contained only general information regarding obesity and associated risks, general advice on healthy eating, and an invitation to practice some PA. | One brief contact, f-up 6, 12 and 24 months |
| Nowicka 2009, Sweden, n=76 | Children referred who are living with obesity | 10.5 (0.95) | 47 | NR | 29.0 (3.1)/ 3.2 (0.5) | One-week summer camp (focusing on PA and healthy dietary behaviours) followed by a 6-months of support with the local sports club. | Sports Coaches  All coaches received training about childhood obesity, motivation, and leadership (3 hrs). | Waitlist control | 27 weeks, f-up- 12 months |
| O’Connor 2013, US, n=40 | 5-8 yrs with overweight (BMI 85%), but not morbid obesity (BMI < 99%); attended participating Texas Children’s Paediatric Associate clinics; and were Texas Children’s Health Plan members | 6.8 (1.1) yrs | 80 | 5 | NR | Promote healthy child lifestyle behaviours with behaviour-specific parenting practices. | TCHP health promotion specialist  Five TCHP health promotion specialists were trained to become Helping HAND health advisors (HAs) during 25 hrs. | Waitlist control | 28 weeks, f-up -7-8 months |
| Olson 2020, US, n=117 | Self-reported Latino descent aged 12-16 years with a BMI at or higher than the 95th percentile for age and sex using CDC growth charts, and prediabetes | 14.0  (1.0) | 40.1 | 0 | 34.0 (5.0)/ 2.0 (0.3) | 1 d/wk of nutrition and health education with behaviour change skills training and 3 d/wk of PA to groups of 8 to 10 families. Set weekly SMART goals. Physical activity was delivered by YMCA instructors twice per week (60 min/session) with unsupervised physical activity promoted for a third day. | Bilingual, bicultural community health educators  NR | After baseline and 6-month measurements, met with a paediatric endocrinologist and a dietitian to discuss laboratory results and develop SMART goals for making healthy lifestyle changes. | 26 weeks, f-up  6 & 12 months |
| Patrick 2013, US, n=101 | 12-16 yrs and at “high risk” for diabetes (i.e, BMI > 85th percentile for age and sex, weight and height >85th percentile, or weight >120% of ideal for height) | 14.3 (1.5) | 63.4 | 17.8 | NR/ 2.2 (0.7) | Focus on education about behavioural goals and promoting use of evidence-based behaviour change strategies.  **Intervention 1**  -Program website with tutorials to promote weight loss and healthy behaviours to include  weekly “check-in” emails, monthly mailed tip sheets and access to the program website and its web tutorials.  **Intervention 2**  -Access to the program website and its web tutorials, monthly mailed tip sheets, and monthly 90 min group sessions of 5–10 adolescents and their parents. Received brief (~20 min) bimonthly phone calls from the health counsellor reviewing concepts presented in the web tutorial  **Intervention 3**  - Program website and its web tutorials, monthly mailed tip sheets, and a minimum of three text messages per week that related to weekly challenges and intervention goals. | Health coaches  NR | Usual care: given printed materials produced by the American Diabetes Association and the American Heart Association, encouraged to attend three 1 h group nutrition sessions during the first 6 weeks at no charge and received monthly tip sheets by mail. | 6 months, f-up, 6 and 12 months |
| Prado 2020, US, n=280 | Hispanic adolescent, was a student in the 7th/8th grade, had a BMI ≥85th percentile adjusted for age and sex, lived with an adult primary caregiver willing to participate | 13.01 (0.8) | 52.3 | NR | NR | 12-wk intervention with 2.5-hr group sessions (8 total) and 1-hr family sessions (4 total), each taking place once a week. During the 4-family sessions, facilitators met individually with each family to practice the skills the parent had learned during the group sessions. | Bilingual facilitators  Had training in using problem-posing, participatory learning, which includes encouraging parents’ active discussion in group sessions. | Usual care: referred to their local health department’s health initiative internet page and the usual programs they offer. | 12 weeks, f-up - 6,12 & 24 months |
| Raynor 2011, US, n=101 (trial 1), 81 (trial 2) | 4-9 yrs, 85th percentile for BMI as determined by the Centres for Disease Control growth charts, and having no  Dietary/PA restrictions | 7.2 (1.7) yrs (Trial 1), 7.1 (1.5) yrs (Trial 2) | 61.4 (Trial 1), 60.5 (Trial 2) | 86.1 (Trial 1), 90.1 (Trial 2) | NR  2.32 (0.64) (Trial 1), 2.27 (0.60) (trial 2) | **Trial 1**:  Intervention 1: monthly newsletters with info about healthy eating and leisure time behaviours + DECREASE: reduced intake of sweet and salty snack foods and sugar-sweetened beverages  Intervention 2: monthly newsletters with info about healthy eating and leisure time behaviours +INCREASE: consume two servings per day of whole fruit, three servings per day of vegetables and two servings per day of low-fat dairy products  **Trial 2**:  Intervention 1: monthly newsletters with info about healthy eating and leisure time behaviours + TRADITIONAL: 60 mins of PA (parents 30 mins) of moderate-intensity PA most days of the week  Intervention 2: monthly newsletters with info about healthy eating and leisure time behaviours + SUBSTITUTES: watch less than or equal to two hours of TV per day and to consume two servings of low-fat milk per day.  Delivered in small groups and consisted of biweekly meetings for 2 months, and then monthly meetings for months 3–6, for a total of eight meetings, with each meeting lasting 45 mins. | Experienced research-staff therapist (either master or doctoral  level) with expertise in nutrition or exercise science, and behaviour modification.  NR | Usual care: monthly newsletters with info about healthy eating and leisure time behaviours. | 6 months, f-up 6 & 12 months |
| Reihner 2010, Germany, n=66 | Children aged 8-16 yrs with overweight (a BMI >90th percentile and <97th percentile using German percentiles), apparently healthy and not on any medication, and attending a regular school | 11.5 (1.6) | 61 | 89 | 23.8 (1.6)/ 1.7 (0.2) | PA training, nutrition education, and behaviour counselling. Interventions were performed in group sessions and individual counselling for the child and their family. 37 sessions for children, 6 sessions for parents, and 5 sessions for parents and children were offered with a total expenditure of 67 h for each family. An interdisciplinary team of paediatricians, diet-assistants, psychologists, and exercise physiologists was responsible for the training. | Paediatricians, diet-assistants, psychologists, exercise physiologists  NR | Waitlist control: received the intervention after 6 months. | 26 weeks, f-up - 6 months |
| Resnicow 2015, US, n=645  (Cluster RCT) | 2-8 yrs with a BMI > 85th and <  =97th percentile based on CDC cutpoints | 5.1 (NR) | 57 | 60 | NR | Intervention 1: 3 MI sessions with parent of child in Year 1 and one additional booster visit in Year 2.  Intervention 2: Same as above but with added counselling from trained registered dieticians (6 MI counselling sessions over 2 years).  Intervention 1: 1.5 days of training in MI and Behaviour Therapy and MI booster training DVD  Intervention 2: same as above but RDs received 1.5 days of MI and BT training, and the MI DVD. | Primary care providers and registered dieticians  NR | Usual care: BMI percentile measured at baseline, 1-year, and 2-year follow-up with routine care by the PCP. | 2 years, f-up, 1 & 2 yrs |
| Resnicow 2024, US, n=1120  (Cluster RCT) | Ages 3 to 11  BMI value for age and sex >85th percentile  >1 health supervision visits with a study clinician in  past 2 years | 7.9 (2.7) | 51.1 | 67.5 | 20.7 (3.8)/NR | 4 key components: in-person and telehealth MI counselling by the pediatric clinicians; telephone MI counselling from a RD; text messages with reminders and tailored behavioural messages; and study portal for RDs, pediatric clinicians, and parents.  All MI sessions centred on counselling on discrete behaviours that have been shown to affect children’s weight. | Primary care providers and registered dieticians    Twelve providers (from 9 intervention practices) received 2 days of in-person training in MI. MI booster training sessions were provided for pediatric clinicians (optional) and required for RDs. | Usual care | 2-2.5 years, f—up, 12, 24 months |
| Sacher 2010, UK, n=116 | 8-12 yrs with a BMI ≥ 98th percentile, no apparent clinical problems, comorbidities, physical disabilities, or learning difficulties, had at least one parent/carer who was able to attend each of the program sessions | 10.3 (1.3) | 54 | 50 | 27.2 (4.3)/ 2.8 (0.6) | Multicomponent healthy lifestyle program including psychology, learning, and social cognitive theories and the study of therapeutic processes. 18, 2-hr group sessions delivered over 9 wks to groups of 8–15 children and their accompanying parents or carers and siblings in community settings. After end of programme, free-family access to a local community swimming pool was made available for a further 12 wks. | Health, social, education, and exercise professionals.  4 days of training and provided with identical materials that detailed methods for the delivery of all sessions. | Waitlist control | 6 months, f-up - 6 & 12 months |
| Saelens 2002, US, n=44 | 12-16 yrs, 20% to 100% above the median (50th percentile) BMI for sex and age,  interested in weight control but not currently engaged in another weight control program, and otherwise healthy as determined by a paediatrician | 14.2 (1.2) | 40.9 | 70.5 | 30.7 (3.1)  /NR | Computer based programme to assess eating, PA and SB and to develop an individualized action plan. Weekly then biweekly phone counselling that lasted 10-20 mins, lasting a total of 14-16 wks. Manuals to help ppts develop behavioural skills for weight control distributed. Self-monitor food and beverage intake. Phone counsellors helped ppts reduce their kcal to ~1200-1500/day and achieve 60 mins of PA on five days/week. | Paediatricians and health counsellors  Received weekly supervision in the provision of behavioural weight control treatment by the first author. | Enhanced usual care: paediatricians instructed to assess/encourage adolescent’s motivation for weight-related behaviour change, provide information about health consequences of high weight status and benefits of better weight control, make recommendations for healthful eating, and PA. | 16 weeks, f-up - 4 & 7 months |
| Sauder 2018, US, n=62 | 7-10 yrs, Tribal members, living with overweight/obesity (BMI ≥85th percentile for age and gender),  ≥1 primary caregiver willing to actively participate | 9.2 (1.1) | 48.5 | NR | 26.2 (4.6)/ 2.65 (1.3) | Active Learning group classes (10 group classes lasting 2 hrs each over a 4-month period in the fall, with two booster classes held in the spring), youth/caregiver dyad MI sessions (5 individual MI counselling sessions and a resource toolbox to facilitate goal attainment, and addressed the individual, home and family, school, community and the health care system domains). | Health coaches  1.5-day workshop with ongoing support available as needed. | Three 1-hr group classes that covered general health and safety topics and each included a healthy group meal. | 6 months, f-up-  8 months |
| Skjåkødegård 2022, Norway, n=114 | BMI above the IOTF cut-off for severe obesity (≥35 kg/m2) or for obesity (≥30 kg/m2) in the presence of weight-related comorbidities | 12.6 (3.1) | 59 | NR | 31.8 (4.9)/ 2.9 (0.4) | Family-based with 17 sessions of structured cognitive behavioural treatment focused on promoting healthy lifestyle behaviours and attitudes using a combination of behavioural and cognitive techniques. | Paediatrician, nutritionist, physiotherapist, nurse and psychologist  All team members were trained in family-based behavioural social facilitation treatment prior to treatment delivery. | Usual care: lifestyle intervention targeting the child with a personalized plan for changing specific lifestyle behaviours and advised to participate in monthly counselling sessions with their local healthcare nurse. | 52 weeks, f-up - 6, 12 & 24 months |
| Small 2014, US, n=60 | Parents of 4-8 yr olds with overweight or obesity | 5.58 (1.43) | 60 | NR | 21.24 (3.25)/ NR | 4 face-to-face sessions (30-60 mins) spaced 4 to 6 wks apart that were based on MI to collaborate with parents on identifying specific realistic healthy lifestyle goals, developing clear steps to reach those goals, routinely having the parents re-evaluate progress, and identifying new goals as needed. Phone calls were provided 2 wks between session. | Trained research assistants  NR | Encouraged to make health and safety goals for their family. | 16 weeks, f-up - 16 wks, 3 & 6 months |
| Smith 2021, US, n=240 | 6 to 12 yrs (changed to 5.5 yrs due to recruitment issues) and elevated BMI (≥85th percentile for age  and gender) | 9.5 (2.0) | 49 | 75 | 26.1 (5.4)  /NR | Family Check-Up 4 Health, assessment-driven individually tailored intervention designed to pre-empt excess weight gain by improving parenting skills. | Trained co-ordinators  Online training, a 3-day in-person small-group training, and one-on-one and group consultation prior to and following delivery to their first two families. | Usual care: received information about the same community resources offered to families in the FCU4Health arm and continued to receive usual care from their providers with frequency of visits determined by BMI classification and progress toward weight management goals. | 6 months, f-up- 3, 6 & 12 months |
| Stark 2011, US, n=18 | 2- 5 yrs with a BMI in the ≥95th percentile but not more than 100% above the mean BMI who had at least one parent with a BMI ≥25, had medical clearance from the child’s paediatrician | 4.6 (1.0) | 70 | 85 | NR/ 2.3 (0.5) | 2-part intervention that targeted lifestyle behaviour modification and improving parenting skills: Phase I (Intensive Intervention): 12 weekly sessions, alternating between group-based clinic sessions (parent and child concurrent groups), and individual home visits  Phase II (Maintenance): 12 wks of every-other-week sessions, alternating between group clinic, and individual home sessions. | Psychology postdoctoral fellows and a research coordinator  NR | Enhanced standard of care: paediatrician counselling delivered in one 45-min visit that delivered dietary and physical activity recommendations outlined by the American Academy of Paediatrics | 24 weeks, f-up- 6 and 12 months |
| Stark 2014, US, n=42 | 2- 5 yrs with a BMI in the ≥95th percentile but not more than 100% above the mean BMI who had at least one parent with a BMI ≥25, had medical clearance from the child’s paediatrician | 4.12 (1.02) | 33.3 | 83.3 | NR | 2-part intervention that targeted lifestyle behaviour modification and improving parenting skills: Phase I (Intensive Intervention): 12 weekly sessions, alternating between group-based clinic sessions (parent and child concurrent groups), and individual home visits  Phase II (Maintenance): 12 wks of every-other-week sessions, alternating between group clinic, and individual home sessions. | Licensed clinical psychologist, psychology postdoctoral fellows and research coordinators  NR | Enhanced standard of care: paediatrician counselling delivered in one 45-min visit that delivered dietary and physical activity recommendations outlined by the American Academy of Paediatrics. | 24 weeks, f-up - 6 and 12 months |
| Stark 2018, US, n=151 | 2-5 yrs, BMI percentile for age and sex ≥95th but no more than 100% above the median BMI, medical clearance from their paediatrician, active patient with anthropometric measurements  within the previous year and living within 50 miles from the medical centre | 55.14 (11.19) months | 57 | 76.2 | NR/ 2.44 (0.6) | 18-session clinic and home family-based behavioural weight management intervention, 3-month intensive treatment phase (weekly sessions) followed by a 3-month maintenance phase (every other week sessions). Intervention sessions alternated between clinic (10 sessions) and home (8 sessions) visits.  Motivational interviewing was a parent only intervention consisting of 18 sessions over 6 months, delivered weekly during the initial 3 months and every other week months 4-6. | Psychologist  Paediatrician and psychologist | Informed caregivers of their child’s weight status during the recruitment process, but neither the children nor caregivers received any treatment. | 6 months, f-up - 6 months |
| -Taveras 2011 & Rifas-Shiman 2016, US, n=475  (Cluster RCT) | 2.0-6.9 yrs, receiving paediatric care at Harvard Vanguard Medical Associates BMI in the 95^th^ percentile or higher or whose BMI was in the 85^th^ to less than 95^th^ percentile if at least 1 parent was living with overweight | 4.9 (1.2) | 48 | 57 | 19.2 (2.4)/ 1.85 (0.63) | Based on the Chronic Care Model and involved changes to the health care system (e.g., enhancing electronic medical record system to assist clinicians with decision support and patient tracking). 4, 25-min chronic diseases management visits and 3, 15-min telephone calls that used MI. Small incentives to further support behaviour change. | Trained the primary care paediatricians in the intervention practices to use brief, focused negotiation skills at all routine well-childcare visits to endorse family behaviour change. | Current standard of care offered by their paediatric practice. | 2 years, f-up- 1 and 2 years |
| Taveras 2015, US, n=549  (Cluster RCT) | 6.0-12.9 yrs, BMI ahealtht the 95th percentile or greater for age and sex, and receipt of well-childcare at Harvard Van guard Medical Associates within the 15 months before enrolment | 9.8 (1.9) | 46.8 | 51.4 | 25.8 (4.3)/ 2.06 (NR) | Clinical Decision Support: paediatric clinicians received decision support on obesity management, and patients and their families received an intervention for self-guided behaviour change. Clinical Decision Support + coaching: decision support was augmented by individualized family coaching - families were assigned a health coach who used motivational interviewing to support families by telephone at 1, 3, 6, and 9 months. Parents were also invited to participate in an interactive text message program where texts were sent twice weekly that supported behaviour change. | Paediatric clinicians and health coaches  Trained to use brief MI | Usual care: Current standard of care offered by their paediatric office. | 52 weeks, f-up- 12 months |
| Taylor 2015, New Zealand, n=206 | All families with children 4 to 8 yrs identified as living with overweight or obesity BMI ≥ 85th percentile) enrolled at 9 general practices or attending secondary care clinics at 1 hospital | 6.5 (1.4) | 55 | NR | 1.6 (0.5) | Single 1-2 hr multidisciplinary consultant session (usually with both parents, mentor dietician, exercise specialist and clinical psychologist) targets for change identified that were specific to each family followed by monthly (yr 1) and tri monthly (yr 2) regular contact, alternating between face-to-face sessions (30-40 mins) at the university and phone calls (5-10 mins) with a MInT mentor (over the 2-year intervention. | Mentors, dieticians, exercise specialists and clinical psychologist.  NR | Met with a trained researcher at baseline (30 to 40 mins) and 6 months. At baseline, parents received individualised feedback about their child’s diet and activity habits and generalised advice using publicly available resources. | 2 years, f-up - 12 and 24 months |
| vanGrieken 2014, Netherlands, n=637  (Cluster RCT) | Overweight according to the international age and gender specific cut-off points for BMI, parents should have at least basic Dutch language skills | 69.09 (5.18) months | 61.9 | NR | NR/ 1.90 (0.37) | During the well-child visit and in up to 3 additional visits, parents of overweight children were offered healthy lifestyle counselling and tailored information regarding a healthy lifestyle. | Youth Health Care Professionals  A workbook with information on theories of behaviour change and practical examples of interviews with parents, half-day training in MI. | Usual care: the well-child visit, during which parents were offered general information about healthy nutrition and PA. | Nost stated , f-up- 24 months |
| VioletSiwik 2013, US, n=32 | Children in 3^rd^- 5^th^ grades ages 8-11 yrs BMI above the 85th percentile and their parents | 9.6 (0.6) | 50 | NR | 26.6 (5.2)/ 2.09 (0.43) | 12 wkly sessions (three 20-to 30-min increments of check-in, class content and PA). Class content included but was not limited to the following topics: portion sizes, fast food, barriers and facilitators to PA and television watching. | Physicians, family medicine residents  2-day training session - our thoughts about problems impact our feelings; and (2) changing our thoughts about situations can change our emotional and behavioural response. | Waitlist control | 12 weeks, f-up - 3,6,9,12 & 15 months |
| Vos 2012, Netherlands, n=81 | 8-17 yrs with obesity (according to Cole et al.) living in the Hague and in the area around the Hague and referred to a paediatrician | 13.2 (1.95) yrs | 53.2 | NR | NR/ 4.24 (0.65) | Multidisciplinary lifestyle treatment, including medical, nutritional, physical, and psychological counselling. 3 months where children were seen at two separate occasions individually by a dietitian for 45 mins, a child physiotherapist for 45 mins, a child psychologist for 90 mins and a social worker for one occasion for 90 mins. Intensive phase (3 months) with 7 group meetings for the children, 5 separate parent meetings and 1 meeting for parents together with their children and booster phase: 2 yrs. | Dietitian, a child-physiotherapist, child psychologist and social worker  NR | Wait list control - initial advice on PA and nutrition. After 1 yr, the children in the control group were offered to participate in the multidisciplinary cognitive behavioural treatment. | 3 month, f-up- 3 & 12 months |
| Wake 2009, Australia, n=258 | 5-10 yrs attending participating practices for any reason during May 2005 to July 2006, not receiving an ongoing weight management programme and were living with overweight or obesity according to the cut-off points of the International Obesity Taskforce | 7.5 (1.4) | 60.4 | NR | 20.2 (2.1)/ 1.9 (0.5) | 4 standard consultations over 12 wks targeting change in nutrition, PA and SB, supported by purpose designed family materials (16 page “family folder” written at a 12-year-old reading level which included five topic sheets, each targeting one area of behavioural change) | GPs  GPs attended two 2½ hr group training sessions for instruction in the “stages of change” model and training in brief, solution focused, family therapy. | Not clear –limited information about comparator is provided. | 12 weeks f-up - as close as possible to 6 & 12 months after randomisation (mean of 9.0 months and 15.2 months) |
| Wake 2013, Australia, n=118 | 3-10 yrs who had obesity who were not receiving an ongoing weight management programme | 7.29 (2.25) | 45.7 | NR | 22.53 (3.12)/ 2.15 (0.4) | 1-hr tertiary weight management appointment where relevant dietary, PA and family/child lifestyle changes were discussed, and care plans and specific goals were set. Long appointment " (20-40 mins) scheduled followed by 4-8 wkly "standard" sessions (6-20 mins) to review lifestyle and BMI progress, identify and solve problems, and set new goals by using brief solution focused techniques. | Paediatricians and dieticians  Basic obesity management to mirror 'real world'. | Usual care: free to seek assistance from their general practitioner or from any other service. | 52 weeks, f-up - 15 months |
| Waling 2012, Sweden, n=105 | Born between 1995–1998, living near or nearby the city, age and gender adjusted BMI ≥ 25 kg/m² | 10.5 (1.1) | 51 | NR | 23.01 (2.6)/ 2.6 (1.90) | Lifestyle changes regarding food habits and PA. 1^st^ yr of intervention: 14 sessions x 1-2/month. 5 sessions concerned food habits, 4 PA, and 5 sessions on behavioural change as well as working towards personal goals and motivation. Between sessions, participants were  given home assignments related to the theme of the upcoming session. Contact was held through a web platform where the participants could report home assignments or communicate with other participants or leaders between the sessions. | University staff, dietician, paediatric physician and child psychologist  NR | Usual care: one information session. | 52 weeks, f-up -12 months |
| Weigel 2008, Germany, n=73 | 7-15 yrs with overweight (defined as body mass index (BMI)  90th percentile for age and gender) or obesity (BMI 97th percentile), and severe obesity (BMI 99.5th percentile), according to the European Childhood Obesity Group and the German Working  Group on Paediatric Obesity | 11.2 (1.7) | 54.8 | NR | 28.6 (3.5)/ 2.4 (0.5) | 1-yr program, composed of modules for PA, nutritional education, and coping strategies, given in 2 sessions/week. There were also monthly parental meetings and medical supervision including laboratory tests at 0, 6 & 12 months. | Sports coaches, dietitians, and psychologist  NR | Written therapeutic advice from a physician during an outpatient visit at 0 & 6 months in the outpatient clinic and were given medical supervision and  laboratory tests at 0, 6, and 12 months. | 12 months, f-up - 6 & 12 months |
| Williamson 2006, US, n=57 | African-American girls, 11-15 yrs; BMI above the 85th percentile for age and gender  based on 1999 National Health and Nutrition Examination  Study normative data; at least one biological parent with obesity and was willing to participate in the study, adolescent’s family was willing to pay $300 out-of-pocket expenses toward the purchase of the computer worth  $1000 and the family home had electricity and at least one functional telephone line | 13.2 (1.4) | 100 | NR | 36.4 (7.9) NR | Adolescent and parent 4 face-to-face counselling sessions during the first 12 wks to encourage adherence to behavioural principles. Web site provided nutrition education and behaviour modification for adults and adolescents using a family-oriented format. Counselling for behaviour modification was accomplished primarily by asynchronous e-mail communications. Participants were encouraged to send e-mails wkly to their counsellor regarding their progress in the program. | Trained counsellors  Educated on culturally relevant dietary and PA issues, and they incorporated this information into the face-to-face and internet counselling sessions. | Received nutrition education from a registered dietitian but were not prescribed behavioural tasks to yield weight loss. The control condition included education on healthy nutrition and exercise. | 2 years, f-up - 6, 9, 12, 18 & 24 months |
| Wylie-Rosett, 2018, US, n=360 | 7-12 yrs and BMI  ≥85th United States CDC BMI percentile for age and sex | 9.3 (1.7) | 51.4 | NR | NR/ 2.0 (0.4) | Behaviour change intervention (8 skill-building core sessions and monthly post-core support sessions focused on dietary modification and increased PA) | Sports coaches, dieticians and social worker  NR | Quarterly paediatrician visits to address weight management recommendations. | 52 weeks, f-up - 3 ,6, 9 & 12 months |
| Zhu 2025, Australia, n=102 | 7-13 yrs with overweight or obesity (BMI ≥ 85th percentile) | 9.9 (1.87) | 49 | NR | 25.44 (4.76)/1.95 (0.45) | 10-week web-based programme that includes key content and behaviour change strategies and weekly phone calls with health coaches | Health coaches that were dieticians, nutritionists and exercise physiologists | Waitlist control | 10 weeks, 10 weeks (programme end) |
| * Study reports zBMI data from the start of the intervention to 6 months for the two randomized groups combined and to 12 months for the intervention group alone  n; number, SD; standard deviation; BMI, body mass index; yrs, years; F-up, follow-up; US; United States, NR; not reported, hr; hour, SB; sedentary behaviour, PA; physical activity, BCTs; behaviour change techniques, wks; weeks, mins; minutes, FB; family-based, PO; parent only, DM2; Type 2 diabetes, MS; metabolic syndrome, IG; intervention group, N; newsletter, GM; growth monitoring, BC; behavioural counselling, GP; general practitioner, wkly; weekly, CBT; cognitive behavioural therapy, TCHP; Texas Children’s Health Plan, HAs; health advisors, CDC; centre for disease control, YMCA; Young Men’s Christian’s Association, MI; motivational interviewing, PCP; primary care practitioner, RDs; registered dieticians, kcal; kilocalories, IOTF; International Obesity Task Force, FCU4Health; Family Check-Up 4 Health, MInT; Motivational Interviewing and Treatment | | | | | | | | | |


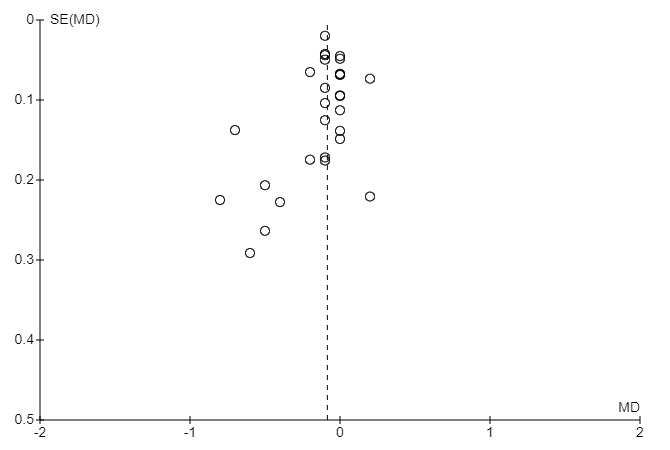


**Figure S1: Funnel plot for zBMI change from baseline to 12 months**

**
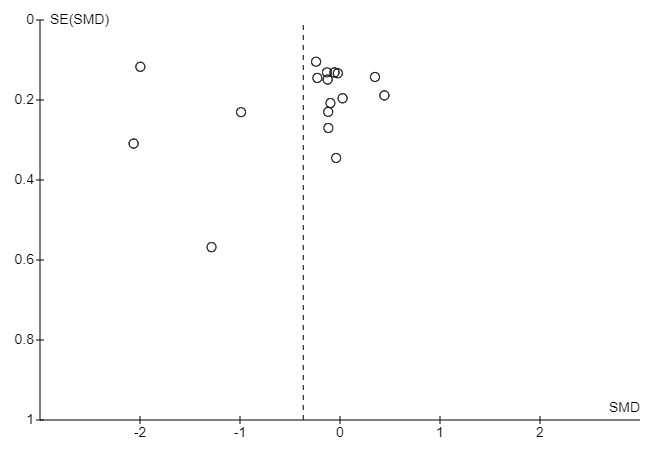
**

**Figure S2. Funnel plot for BMI change from baseline to 12 months**


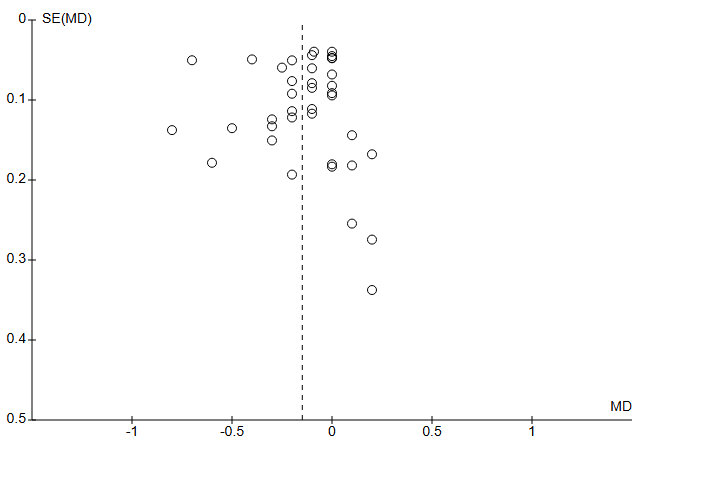


**Figure S3: Funnel plot for zBMI change from baseline to programme end**


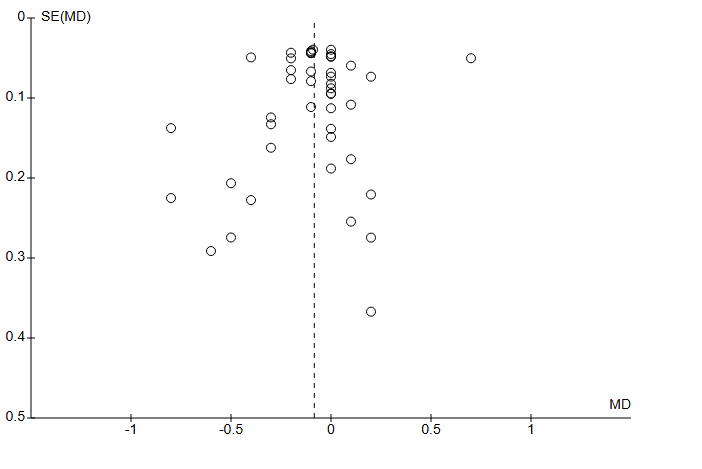


**Figure S4: Funnel plot for zBMI change from baseline to last follow-up**


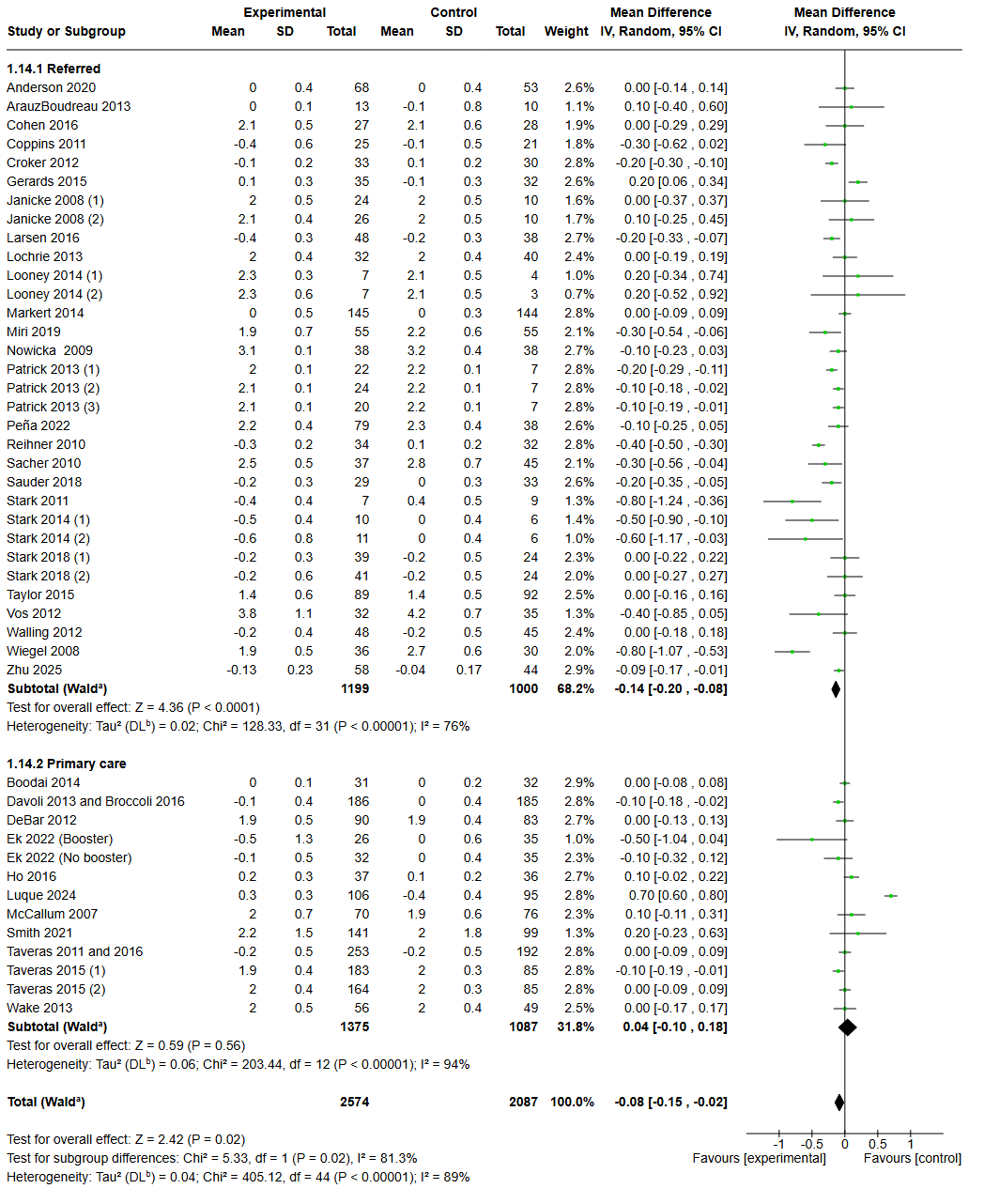


**Figure S5: zBMI change from baseline to last follow-up for interventions delivered in or referred to by primary care**


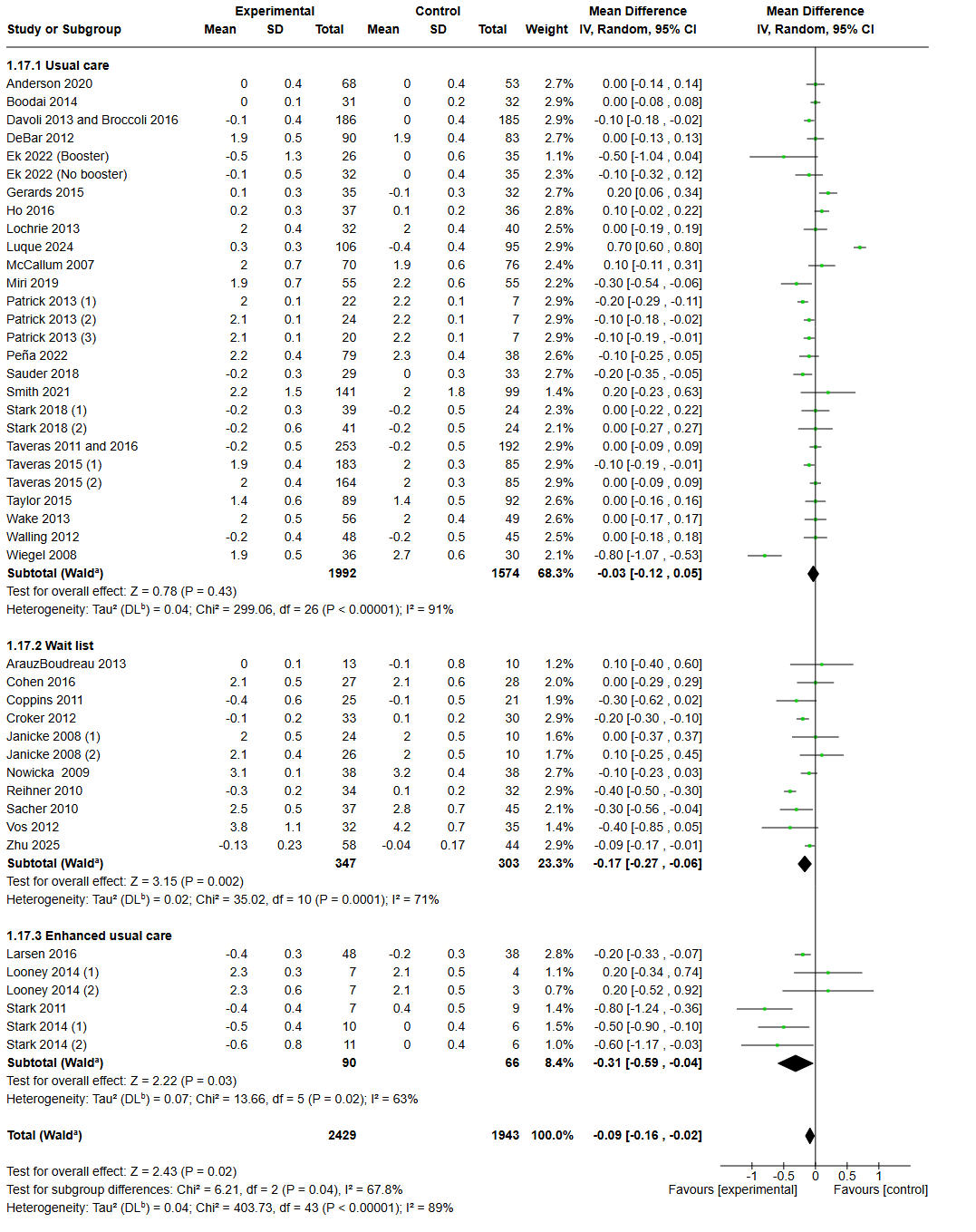


**Figure S6: zBMI change from baseline to last follow-up for studies that compared the effect of the intervention to usual care, a waitlist control or enhanced usual care**


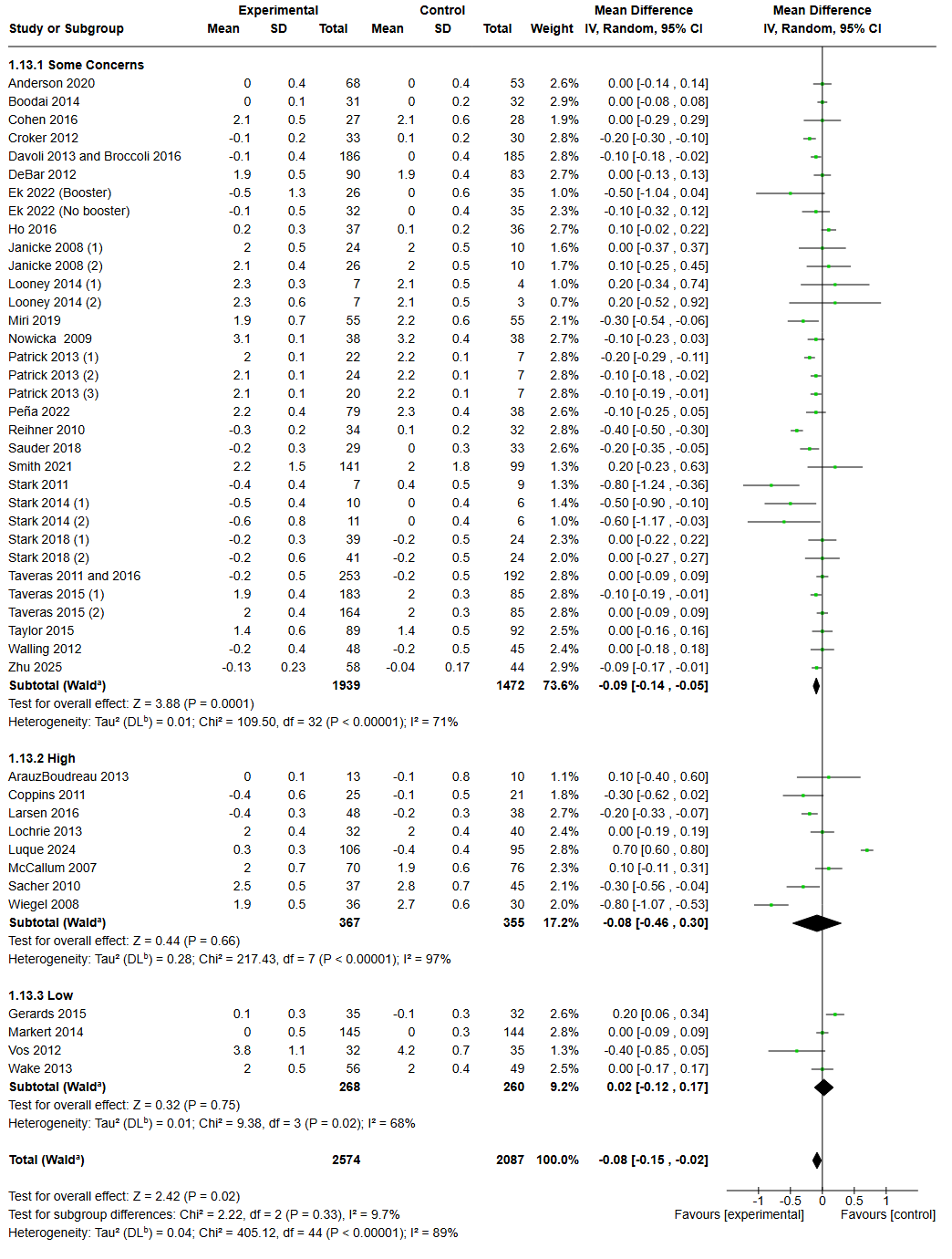


**Figure S7: zBMI change from baseline to last follow-up for studies rated low risk of bias vs some concern for risk of bias vs high risk of bias**


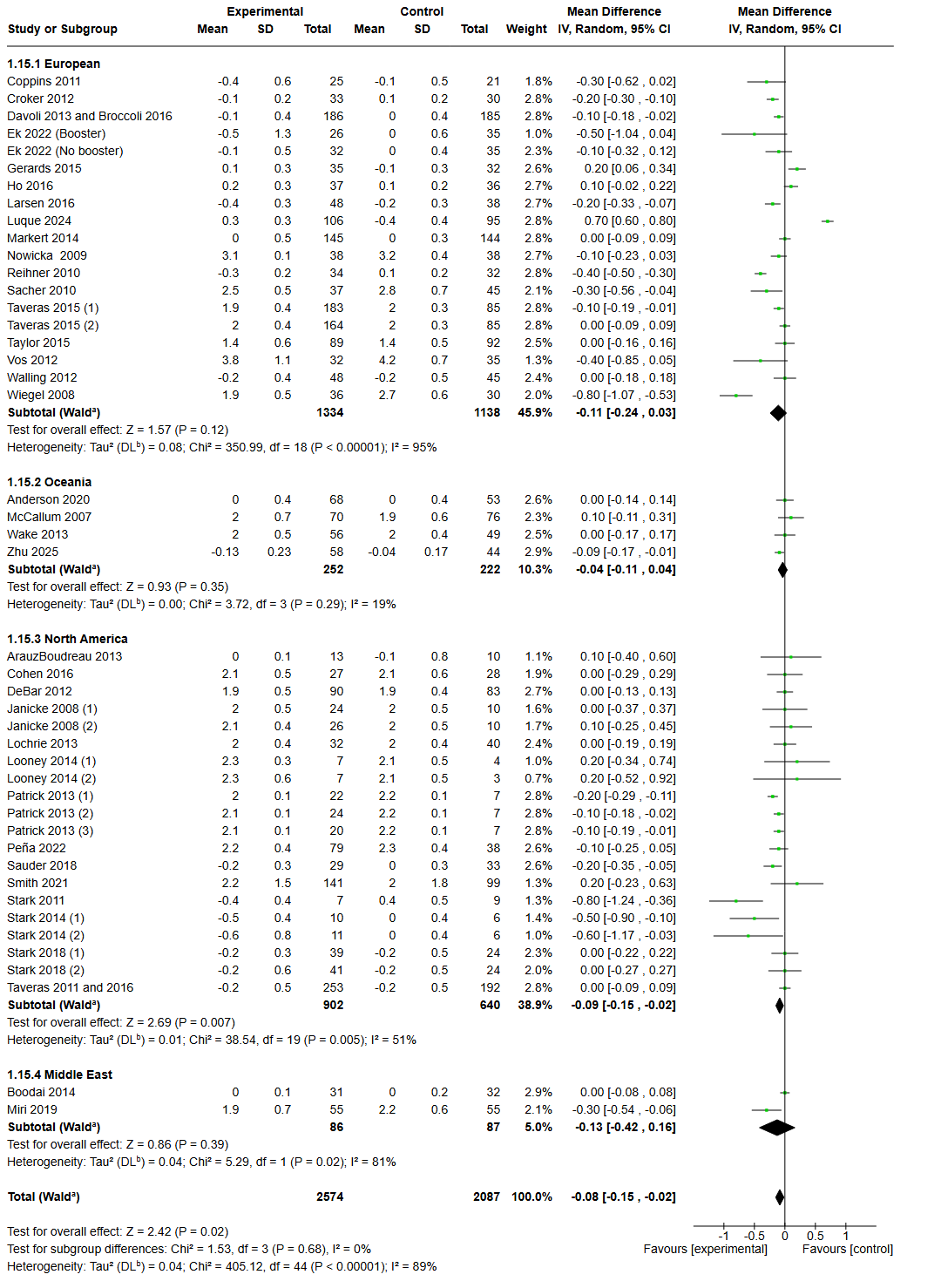


**Figure S8: zBMI change from baseline to last follow-up for interventions delivered in North America, Europe, Middle East and Oceania**


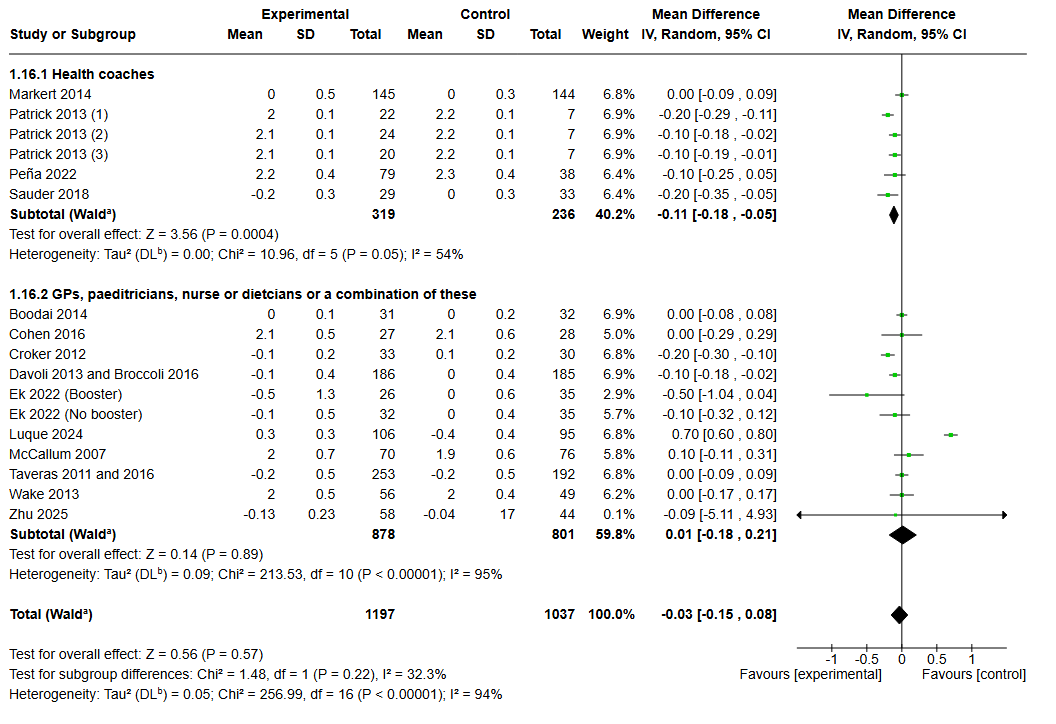


**Figure S9: zBMI change from baseline to last follow-up for interventions delivered by health coaches or GPs, paediatricians, nurses, dieticians or a combination of these**


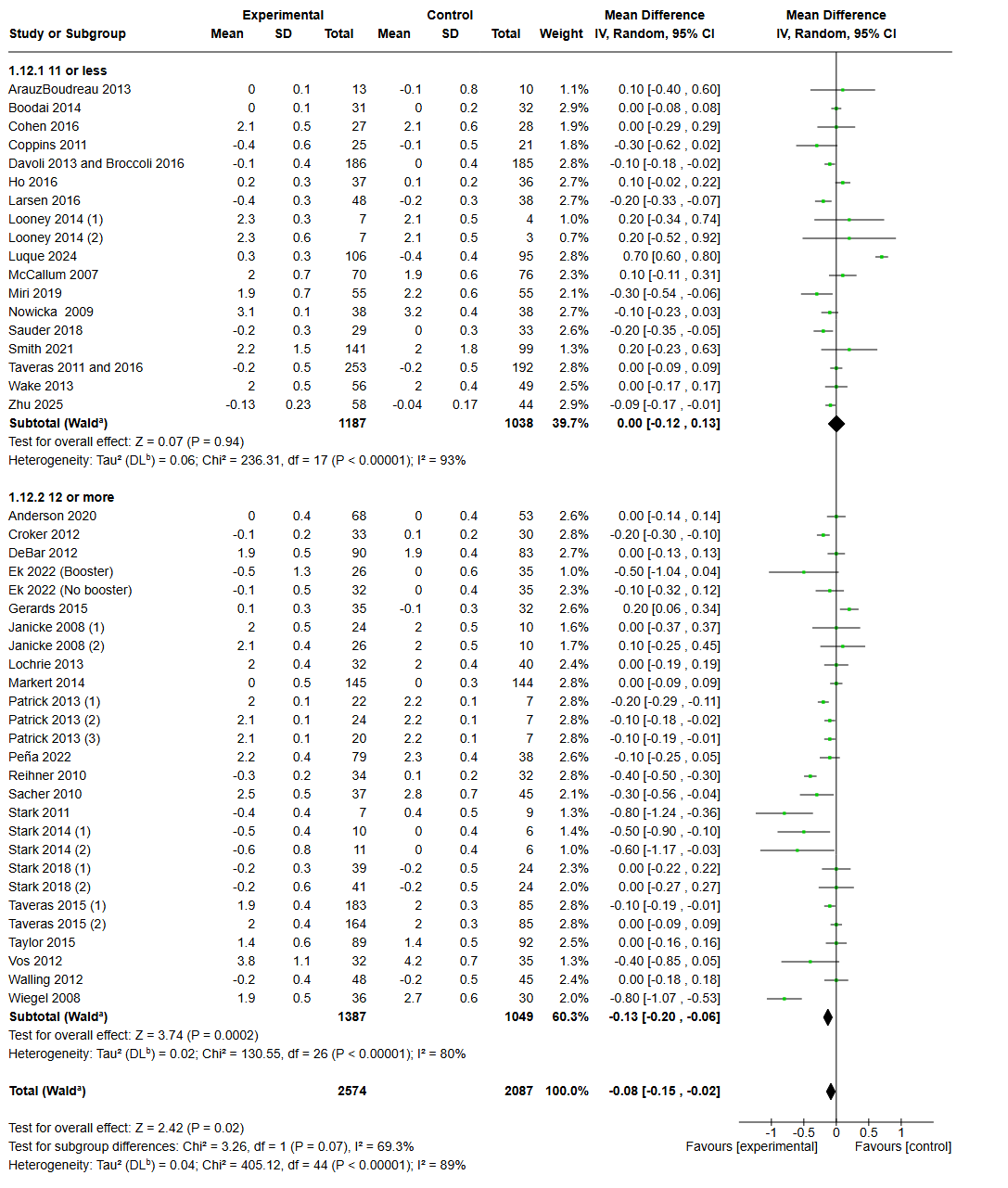


**Figure S10: zBMI change from baseline to last follow-up for interventions with 11 or less contacts and interventions with 12 or more contacts.**
